# Hepatitis C Virus Core Protein Promotes miR-122 Destabilization by Inhibiting GLD-2

**DOI:** 10.1371/journal.ppat.1005714

**Published:** 2016-07-01

**Authors:** Geon-Woo Kim, Seung-Hoon Lee, Hee Cho, Minwoo Kim, Eui-Cheol Shin, Jong-Won Oh

**Affiliations:** 1 Department of Biotechnology, Yonsei University, Seoul, Korea; 2 Laboratory of Immunology and Infectious Diseases, Graduate School of Medical Science and Engineering, KAIST, Daejeon, Korea; The Scripps Research Institute, UNITED STATES

## Abstract

The liver-specific microRNA miR-122, which has essential roles in liver development and metabolism, is a key proviral factor for hepatitis C virus (HCV). Despite its crucial role in the liver and HCV life cycle, little is known about the molecular mechanism of miR-122 expression regulation by HCV infection. Here, we show that the HCV core protein downregulates the abundance of miR-122 by promoting its destabilization via the inhibition of GLD-2, a non-canonical cytoplasmic poly(A) polymerase. The decrease in miR-122 expression resulted in the dysregulation of the known functions of miR-122, including its proviral activity for HCV. By high-throughput sequencing of small RNAs from human liver biopsies, we found that the 22-nucleotide (nt) prototype miR-122 is modified at its 3′ end by 3′-terminal non-templated and templated nucleotide additions. Remarkably, the proportion of miR-122 isomers bearing a single nucleotide tail of any ribonucleotide decreased in liver specimens from patients with HCV. We found that these single-nucleotide-tailed miR-122 isomers display increased miRNA activity and stability over the 22-nt prototype miR-122 and that the 3′-terminal extension is catalyzed by the unique terminal nucleotidyl transferase activity of GLD-2, which is capable of adding any single ribonucleotide without preference of adenylate to the miR-122 3′ end. The HCV core protein specifically inhibited GLD-2, and its interaction with GLD-2 in the cytoplasm was found to be responsible for miR-122 downregulation. Collectively, our results provide new insights into the regulatory role of the HCV core protein in controlling viral RNA abundance and miR-122 functions through miR-122 stability modulation.

## Author Summary

Viruses may benefit from altering the host cell’s normal miRNA milieu, by either lowering or increasing antiviral or proviral miRNA levels. Our results reveal a mechanism by which virus infection can regulate miRNA abundance by modulating the activity of cellular enzymes responsible for miRNA 3′-terminal modifications. Hepatitis C virus (HCV) uses microRNA-122 (miR-122) as a proviral host factor to increase viral genome abundance, but HCV infection results in diminished miR-122 levels by unknown mechanisms. We demonstrated that the HCV core protein plays a role in reprogramming the cellular profile of miR-122 isomers by inhibiting GLD-2, a non-canonical cytoplasmic poly(A) polymerase, to promote miR-122 destabilization. Our results raise several important questions. First, how is only a specific subset of miRNAs in the liver affected by GLD-2 inhibition by the HCV core protein? Second, how does HCV regulate pre-miR-122 processing in its loop region? Third, does GLD-2 inhibition by the HCV core protein affect the translation of a specific group of host mRNAs? With many unanswered questions, our results propose a novel feedback regulatory mechanism for controlling HCV RNA abundance by the viral capsid protein, which is capable of modulating miR-122 activity and stability through the inhibition of GLD-2.

## Introduction

Hepatitis C virus (HCV), a positive-sense single stranded RNA virus, causes chronic hepatitis and liver cirrhosis, often leading to the development of hepatocellular carcinoma. The HCV genome is composed of a long open reading frame (ORF) that is flanked by untranslated regions (UTRs) at both the 5′ and 3′ ends. The ORF encodes a polyprotein of approximately 3010 amino acids that is processed by cellular and viral proteases into 10 polypeptides, including structural (core protein and envelope proteins E1 and E2) and non-structural (NS) proteins [1].

MicroRNA-122 (miR-122) is the most abundant miRNA in the liver [2, 3]. miR-122 binds to two closely spaced target sites in the 5′-UTR of the HCV genome to promote viral RNA stability and accumulation by diverse mechanisms [4–8]. Sequestration of miR-122 with miravirsen, an antisense oligonucleotide targeting miR-122, resulted in a prolonged and dose-dependent decrease in HCV RNA titers in a clinical study [9]. In addition to its proviral function for HCV, miR-122 regulates hepatic function and cholesterol and fatty-acid metabolisms [10, 11]. Interference of miR-122 function using an antisense oligonucleotide decreased cholesterol levels in plasma [11]. In addition, it was found that miR-122 downregulates its target genes that may be involved in tumorigenesis and metastasis, thus acting as a tumor suppressor [12–15].

Functional relationships between viruses and cellular miRNAs are likely to play important roles in viral pathogenesis and in modulating virus replication by either promoting or limiting replication [16, 17], as also studied with HCV using various miRNAs that directly bind to the viral genome [18]. In addition, the interface between viruses and miRNAs can alter predefined cellular regulatory networks of miRNAs by re-programming miRNA types and abundance. miRNA expression is controlled at various stages of transcription and processing, and its level and function can be further regulated by mature sequence modifications. miRNA 3′ modification is known to regulate its stability or turnover [19], and recent high-throughput sequencing studies revealed extensive 3′-terminal modifications on miRNAs in animal cells [20]. Despite the ample evidence of post-transcriptional modification on mature miRNAs, the impact of miRNA 3′-end modifications is largely unknown.

Previous studies illustrated that miR-122 levels are decreased in HCV-infected cells [21]. In addition, a decreased miR-122 level was detected in sera from patients with HCV [21, 22]. However, little is known regarding how the cellular abundance of miR-122 is regulated by HCV. We report in this study that the HCV core protein inhibits GLD-2’s terminal mononucleotidyl transferase activity, resulting in the downregulation of miR-122 isomers bearing non-templated 3′-end single-nucleotide additions and thereby the dysregulation of miR-122 function. Our results demonstrate a novel role of the HCV core protein in regulating viral replication and translation through GLD-2-mediated miR-122 stability control.

## Results

### Downregulation of miR-122 by HCV is attributed to the core protein

**Fig 1:**
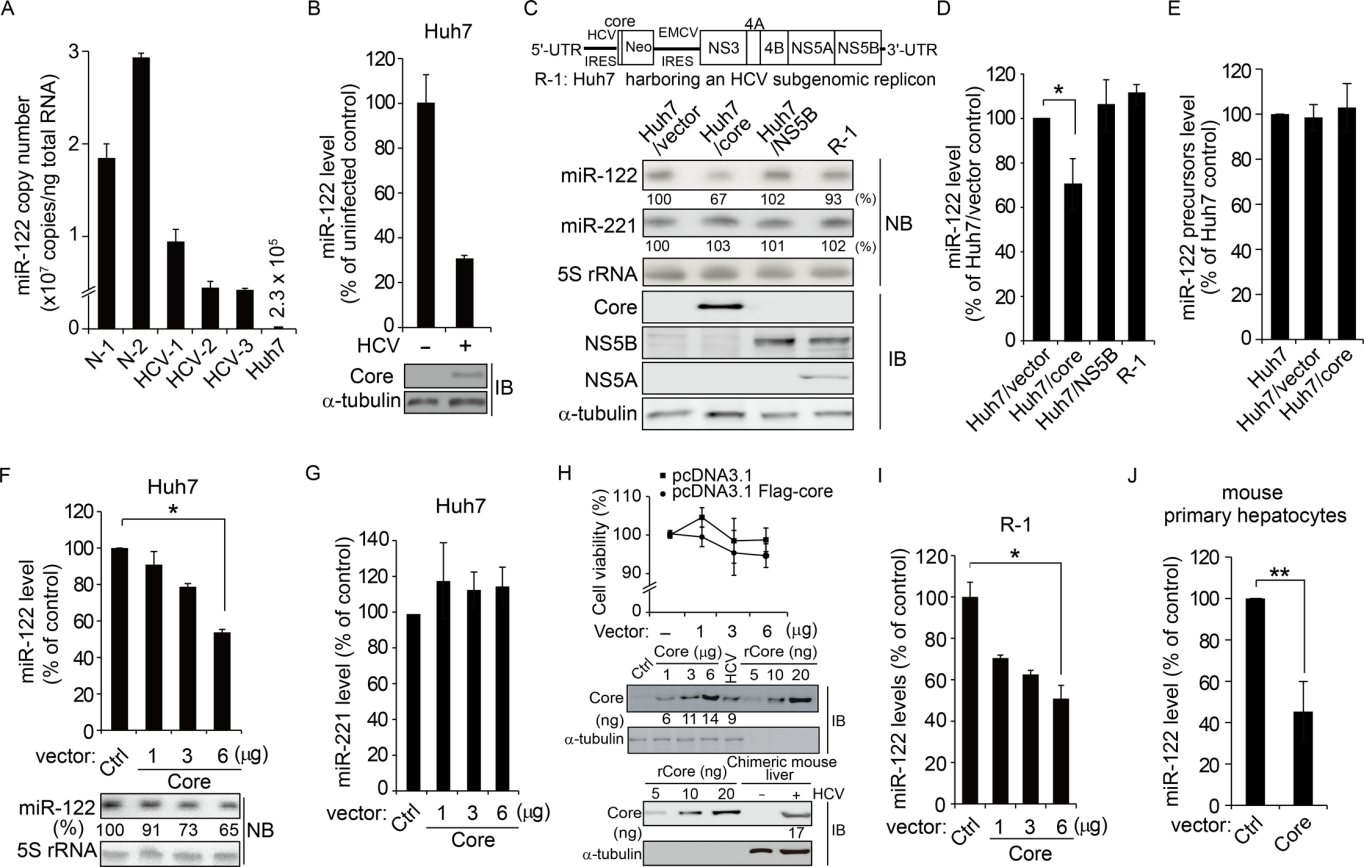
Downregulation of miR-122 expression by HCV core protein. (A) qRT-PCR quantification of miR-122 levels normalized to U6 snRNA in liver biopsies from patients with HCV (HCV-1 to HCV-3) and healthy controls (N-1 and N-2) and in Huh7 cells. Results are expressed as a percentage of the miR-122 level in the normal liver specimen N-1. (B) Relative miR-122 level in Huh7 cells infected with HCV (MOI of ~0.25) at 2 days post infection. Core protein expression was assessed by immunoblotting (IB). (C and D) Levels of miR-122 and miR-211 in the indicated cell lines were analyzed by northern blotting (NB), quantified using a PhosphorImager, and normalized using 5S rRNA (C). The numbers below the lanes indicate the estimated miRNA level compared with control (Huh7/vector cell line). Shown in the bottom panels are the results of immunoblotting (IB) analyses for the indicated proteins in cell lysates. Plotted in (D) are the relative levels of miR-122 (mean ± SD) estimated from three independent experiments. *P = 0.0032. (E) Levels of miR-122 precursors were analyzed by real-time qRT-PCR of total RNAs isolated from indicated stable cell lines. (F–H) Huh7 cells were transfected with pcDNA3.1 (6 μg, Ctrl) or increasing amounts of pcDNA3.1-Flag-core (1, 3, and 6 μg; Core). After 48 h, cells were harvested to assess expression levels of miR-122 (*P = 0.0025; F) and miR-221 (G), and cell viability (H). In (H), total cell lysates (30 μg) from the transfected cells and HCV-infected cells, and liver tissue clear lysates (100 μg) from a chimeric mouse harboring human hepatocytes were resolved by SDS-PAGE along with a purified His-tagged recombinant HCV core protein and analyzed by immunoblotting for quantification of core protein expression levels. (I and J) The miR-122 level in R-1 cells (I) or primary mouse hepatocytes (J) transfected with pcDNA3.1 (Ctrl) or pcDNA3.1-Flag-core plasmid (Core) was determined 2 days after transfection. *P = 0.0042; **P = 0.0114. In (D), (F), (I), and (J), the error bars are the standard deviations of three independent experiments, each involving triplicate assays. Statistical significance of difference between groups was determined via an unpaired Student’s *t*-test.

We analyzed miR-122 expression levels in human liver biopsies from healthy subjects and patients with HCV (S1 Table show the characteristics of the liver biopsies used in this study) and observed decreased miR-122 levels in liver specimens from patients (66 ± 3.7%, 72 ± 0.7%, and 74 ± 1.9% decreases in liver biopsies HCV-1, HCV-2, and HCV-3, respectively from patients infected with HCV) compared with those in the normal liver N-1 (Fig 1A). In the hepatocellular carcinoma cell line Huh7, which is widely used in HCV studies because of its capability to support HCV replication, the miR-122 level was found to be approximately 100-fold lower than that in the normal liver N-1 (1.25% of miR-122 levels in N-1). In this cell line, HCV infection elicited a 70 ± 1.5% decrease in miR-122 levels at 2 days post infection (Fig 1B) when ~30–40% of cells were infected as determined by immunostaining of core protein (S1A Fig). The miR-122 level decreased gradually during the course of HCV infection as early as day 1 post-infection (S1B Fig).

To identify the viral proteins involved in this regulation of miR-122 expression, we monitored miR-122 levels in Huh7-derived cell lines that individually express the HCV core protein, NS5B, and NS proteins (NS3 to NS5B from an HCV genotype 1b subgenomic replicon, which constitutively replicates in the R-1 cell line; Fig 1C, top). We observed a significant decrease in the expression of the mature form of miR-122 when the HCV core protein was expressed (30 ± 11.5% decrease; Fig 1C and 1D), whereas its levels were not influenced by NS proteins. Notably, the expression of miR-122 precursors (primary and precursor forms of miR-122) was not altered by the core protein (Fig 1E), demonstrating that miR-122 transcription and biogenesis were not affected by core protein expression.

In Huh7 cells transiently expressing the HCV core protein, decreased miR-122 levels were also observed, as assessed by real-time quantitative RT-PCR (53 ± 1.6% of an empty vector-transfected control) and northern blot analysis (35% decrease compared with miR-122 levels in the 6-μg empty plasmid-transfected control) (Fig 1F), whereas miR-221 levels were not altered upon core protein expression (Fig 1G). Transient expression of the core protein did not reduce cell viability (Fig 1H). Its expression level in Huh7 cells was in the range of 0.2–0.46 ng/μg total protein. This level was comparable to that (0.3 ng/μg total protein) in Huh7 cells infected with HCV (MOI = 0.25) and that (0.17 ng//μg total protein) in the liver of HCV-infected SCID mouse with chimeric liver repopulated with human hepatocytes (S1C and S1D Fig show the percentage of core protein-positive cells in the chimeric liver and serum HCV RNA titer in the infected mouse, respectively). The downregulating effect of the HCV core protein was also observed both in R-1 cells (Fig 1I) and in mouse primary hepatocytes (Fig 1J), which express the same mature form miR-122 as in human hepatocytes.

### Dysregulation of miR-122 function by the core protein

**Fig 2:**
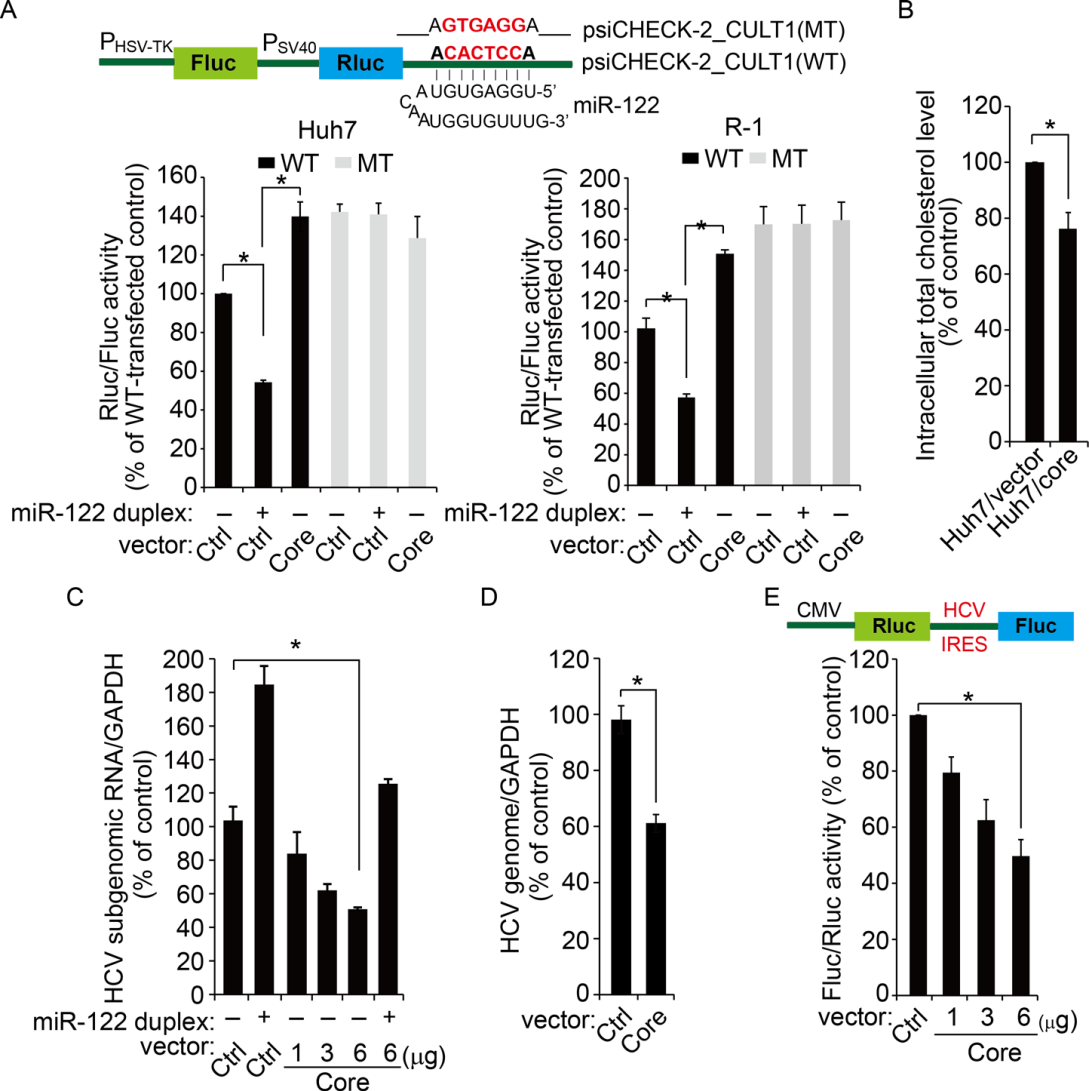
Dysregulation of miR-122 function by the HCV core protein. (A) Schematic representation of a reporter plasmid carrying a miR-122 target site [psiCHEK-2_CULT1(WT)] or a miR-122 mismatched target [psiCHEK-2_CULT1(MT)] at its 3′-UTR (top). Huh7 or R-1 cells treated with miR-122 duplexes were transfected with each indicated reporter plasmid together with pcDNA3.1 (Ctrl) or pcDNA3.1-Flag-core (Core). At 48 h post-transfection, cells were harvested, and normalized luciferase activity (Rluc/Fluc) compared with empty vector-transfected control was measured (bottom). *P < 0.005. (B) Intracellular total cholesterol content. The cholesterol content in core-expressing cells (Huh7/core) was 76.5 ± 5.1% of the Huh7/vector control. *P = 0.0013. (C) R-1 cells transfected with the pcDNA3.1-Flag-core (Core) or empty vector (Ctrl) were treated with miR-122 duplex. The HCV subgenomic RNA titer was determined 2 days after transfection by real-time qRT-PCR, normalized with GAPDH, and expressed as a percentage of that in empty vector-transfected control cells. *P = 0.0032. (D) Huh7 cells pre-transfected with pcDNA3.1 (Ctrl) or pcDNA3.1-Flag-core (Core) plasmids were infected with HCV prior to the quantification of the HCV RNA titer by qRT-PCR 2 days later. *P = 0.0011. (E) Huh7 cells were transfected with increasing amounts of pcDNA3.1-Flag-core (Core) or empty vector (6 μg; Ctrl) together with a dual luciferase reporter expressing *Renilla* luciferase (Rluc) and firefly luciferase (Fluc) by cap- and IRES-dependent translation (top), respectively, before dual luciferase assays at 48 h post-transfection. *P = 0.0022. For all panels, error bars are standard deviations of three independent experiments, each involving triplicate assays. *P* values were calculated using an unpaired *t*-test.

We performed a series of experiments to evaluate the impact of miR-122 downregulation. First, using a psiCHEK-2_CULT1(WT) dual-luciferase reporter vector that contains the CULT1 3′-UTR, a known target of miR-122 [23], we demonstrated that the reporter activity, which was suppressed by miR-122 duplex transfection, could be rescued by the HCV core protein both in Huh7 and R-1 cells (Fig 2A). As expected, this rescuing effect and target gene-suppressing activity disappeared when the miR-122-binding site in the dual reporter was mutated.

miR-122 is involved in the cholesterol synthesis pathway, and the plasma cholesterol level decreases upon the inhibition of miR-122 using an antisense oligonucleotide [10]. Cellular cholesterol metabolism plays direct or indirect roles in the HCV life cycle [24]. We found a decrease in the cellular total cholesterol content in the core protein-expressing stable cell line Huh7/core (Fig 2B), elaborating a recent observation of hypocholesterolemia induced by HCV infection [25] and suggesting potential regulatory role of HCV core protein in cellular cholesterol biosynthesis [10].

HCV RNA abundance is regulated by miR-122 binding to the HCV 5′-UTR [4, 5, 26]. miR-122 transfection into R-1 cells increased HCV subgenomic RNA titer (Fig 2C), confirming the previous findings. As shown in Fig 2C and 2D, the expression of the core protein in R-1 cells and its expression in Huh7 prior to HCV (JFH-1) infection resulted in significant decrease in HCV RNA levels (50.8 ± 2.8% and 61 ± 5.4% of control, respectively). As expected, we observed that miR-122 added exogenously could rescue the inhibitory effects of core protein in R-1 cells.

Furthermore, using a dual luciferase reporter system that expresses the *Renilla* luciferase reporter (Rluc) in a cap-dependent manner and firefly luciferase (Fluc) reporter by HCV internal ribosome entry site (IRES)-mediated initiation, we were able to demonstrate the gradual inhibition of HCV IRES-mediated translation with an increase in the HCV core protein expression (maximum 51 ± 5.9% decrease compared with 6-μg empty vector-transfected control; Fig 2E).

### Alteration of miR-122 isomer profile by HCV infection

Recent studies illustrated that post-transcriptional 3′-terminal modification of the mature or precursor forms of miRNAs can regulate their turn-over by promoting either stabilization or destabilization [20, 27, 28]. We intended to test the possibility that HCV infection affects this miRNA modification process to alter the profile of miR-122 isomers. Therefore, we performed high-throughput sequencing of small RNA libraries from human liver biopsy specimens from healthy individuals (N-1 and N-2) and patients with HCV (HCV-1 to HCV-3) (S2 Table shows details on the small RNA types and their read counts). Among diverse miRNA [578 (for HCV-2) to 775 (for HCV-3) species identified by aligning the reads to 1,080 prototype miRNAs in the miRNA database] expressed in human liver tissues, miR-122 was the most abundantly expressed miRNA. We detected various miR-122 species in the range of 16 to 25 nucleotide (nt) in length (S3 Table). Because miR-122 isomers of 21, 22, and 23 nt in length are three prominent species, which were detectable by northern blot analysis of total RNA from Huh7 cells and primary hepatocytes (S2A and S2C Fig), and other individual isomers comprised <2% of the total read counts of all forms of miR-122 species (with a read frequency of >50 reads per million) in biopsies, we focused on these three isomers for further sequence analysis.

**Fig 3:**
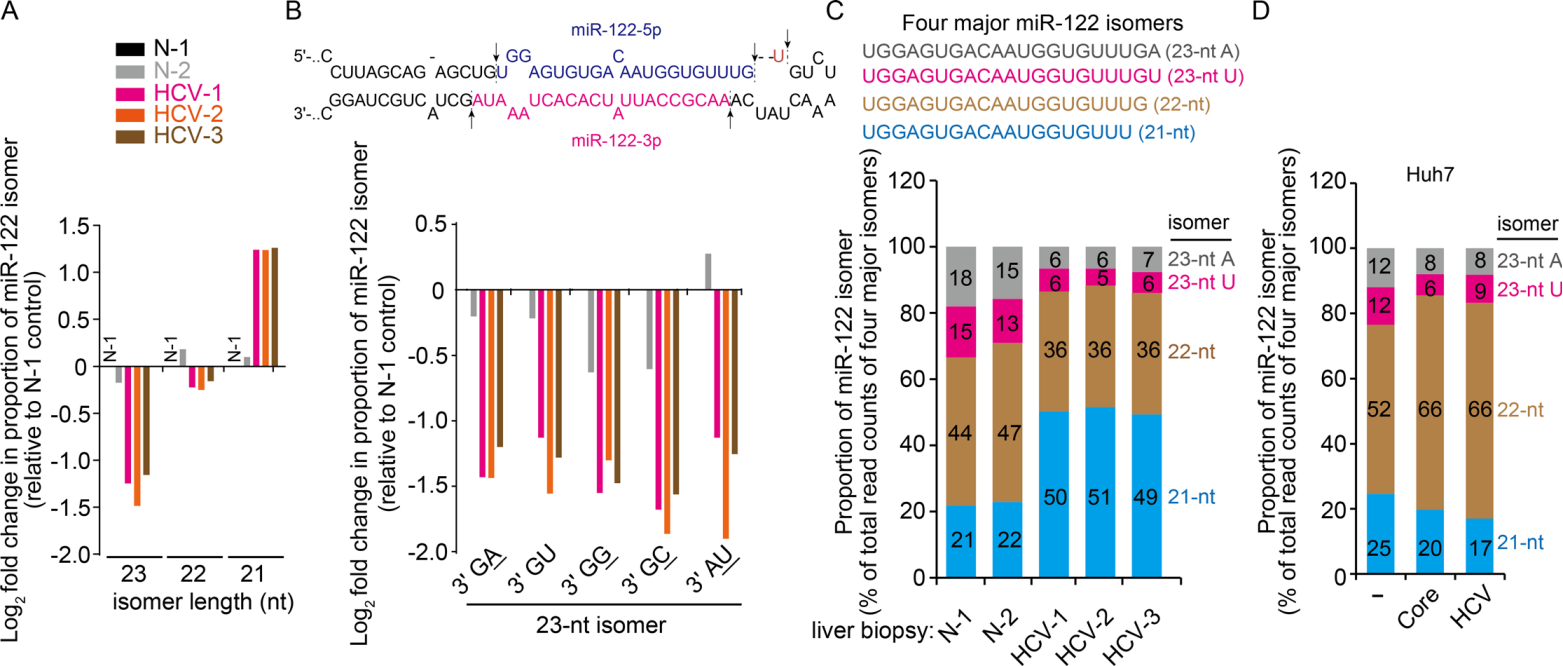
Non-templated nucleotide addition to the 3′ end of miR-122 is suppressed in liver tissue from patients with HCV and in Huh7 cells expressing HCV core protein or infected with HCV. (A) Analysis of miR-122 isomers identified by the deep sequencing of small RNA libraries from human liver biopsies. Fold change (log2) of the proportion of each miR-122 isomer (each isomer read count divided by the total count of miR-122 isomers) in the indicated specimens compared with the corresponding value in the normal liver tissue (N-1). (B) Fold-change analysis for the 23-nt isomers as described in (A). Isomers are sorted according to their 3′-end dinucleotide sequences. Presented at the top is the pri-miR-122 sequence showing processing sites (blue arrows) for the 22-nt prototype miR-122 and an aberrant 3′-terminal processing site (black arrow) for the 23-nt isomer bearing a template-derived 3′ GU-tail. (C) Proportions (% of each isomer read count compared with the total read counts of the four major isomers shown at the top) of four major miR-122 isomers (isomers whose relative percentage to the total read counts of miR-122 isomers is >2%) in the indicated liver biopsies. (D) Proportions of four major miR-122 isomers in Huh7 cells transiently expressing HCV core protein (Core) or infected with HCV.

Interestingly, the proportion of individual miR-122 isomers was substantially altered in patient liver biopsies. As shown in Fig 3A, there was a >2-fold increase in the proportion of the 21-nt miR-122 isomer compared with that in the normal liver N-1. In contrast, the proportion of the 23-nt isomer dramatically decreased, whereas the ratio for the 22-nt miR-22 prototype species only slightly decreased. The 23-nt isomer, including five different species, had either a templated nucleotide [i.e., the 3′-end U residue derived from precursor miR122 (pre-miR-122); Fig 3B, top shows the partial primary miR-122 (pri-miR-122) secondary structure and sequence] or a non-templated nucleotide (i.e., isomers bearing a dinucleotide of 3′-GA, 3′-GG, and 3′-GC in the 23-nt isomer in which the penultimate G is derived from a template or a 3′-AU dinucleotide; underlined sequences represent non-templated additions) at their 3′ ends. In addition to the 3′-end mono-adenylated isomer, we could also detect miR-122 isomers carrying a 3′-mono-G or -C tail in the liver biopsies, but these isomers represented <0.2% of the total read counts of all miR-122 species (S3 Table). In fact, isomer profile analysis for the top 50 most abundantly expressed miRNAs in the liver biopsies revealed that all miRNAs bear a single 3′ terminal non-templated or templated nucleotide (any of four ribonucleotide residues) with adenylate and uridylate being two major terminal nucleotides (S3 Fig).

Notably, in patient liver specimens, all of the miR-122 isomers with a non-templated nucleotide added to the 3′-end of 22-nt prototype miR-122 exhibited a similar decrease in their proportions (Fig 3B). In particular, the proportion of two major 23-nt isomers, namely the A-tailed and U-tailed isomers, was decreased by approximately 2-fold in HCV-infected liver samples (Fig 3C) compared with that in normal liver tissue specimens (N-1 and N-2). Similar results were also observed both in Huh7 cells transiently expressing core protein and in HCV-infected Huh7 cells (Fig 3D), in which only 3′-end mono-adenylated or uridylated species were detected probably due to ~100-fold lower miR-122 levels in Huh7 cells (Fig 1A and S4 Table).

We found that HCV-mediated inhibition of 3′-end monoadenylation and uridylation is limited to a specific set of miRNAs in the liver (S4 Fig). Analysis of small RNA sequencing datasets for each species of the 24-nt miR-122 isomers (four different species of 24-nt isomer harboring an AA, AU, UU, and UA dinucleotide; underlined sequences represent non-templated nucleotides) also revealed that their relative ratios decreased by >2-fold in HCV-infected liver tissues, although the total read counts of the 24-nt long miR-122 isomers was relatively low (approximately 1% of total reads; S3 Table). The results together demonstrate that regardless of the types of 23- and 24-nt miR-122 isomers bearing a single nucleotide or dinucleotide, their relative proportions were decreased in liver tissues from patients with HCV. In particular, the non-templated addition of adenylate residues, which is the major modification event of miR-122, was substantially inhibited by HCV.

### HCV core protein inhibits GLD-2, which specifically regulates the cellular abundance of miR-122

**Fig 4:**
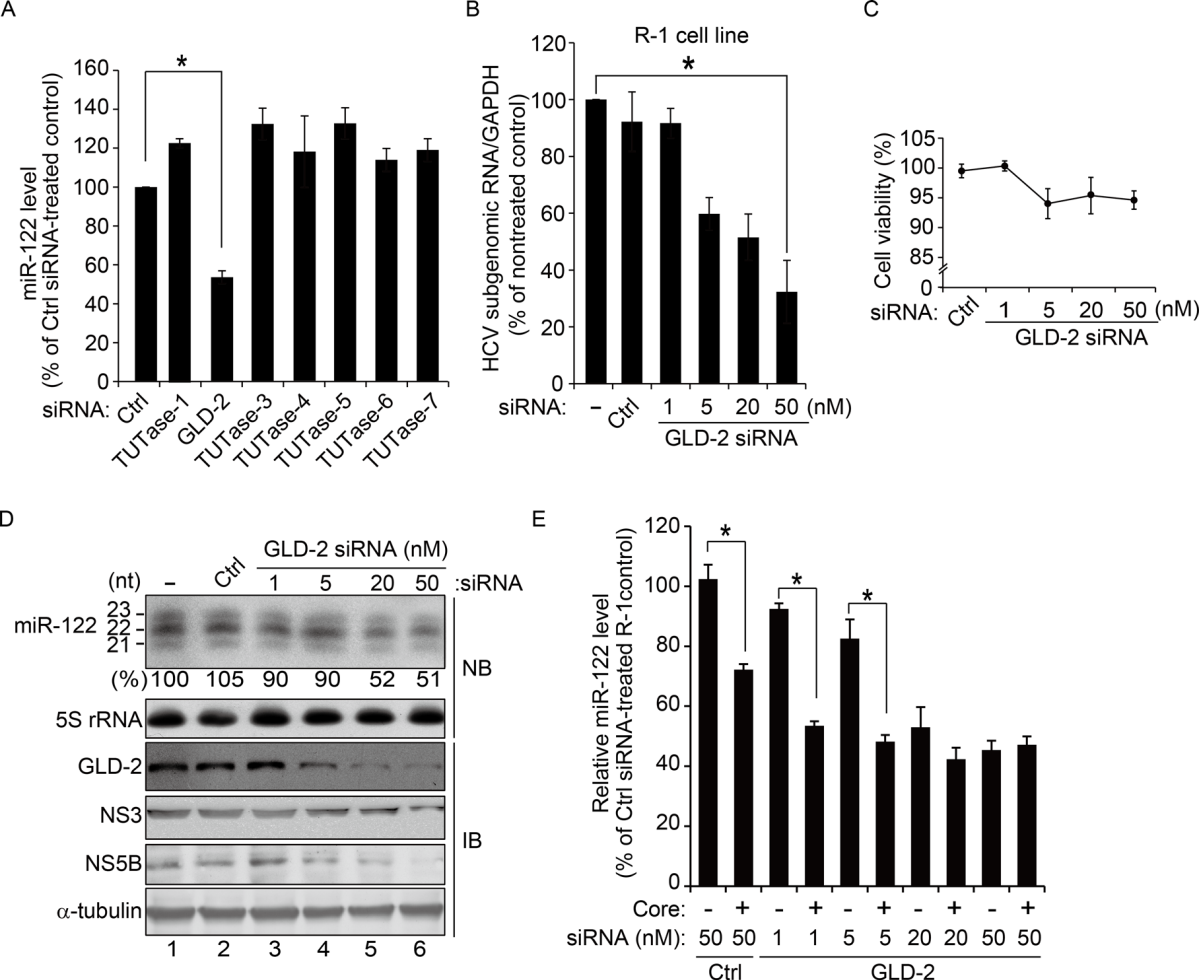
Cellular abundance of miR-122 is specifically regulated by GLD-2. (A) mir-122 level in Huh7 cells silenced for indicated terminal nucleotidyl transferases using siRNA. The miR-122 level was determined by real-time qRT-PCR at 48 h post-siRNA transfection (20 nM). Ctrl, scrambled siRNA. *P = 0.0029. (B–D) HCV subgenomic replicon RNA titer (B), cell viability (C), and miR-122 level (D) in R-1 cells treated with increasing concentrations of GLD-2 siRNA. Shown in (B) are the relative viral RNA levels in GLD-2-silenced cells compared with that in non-treated control cells at 48 h post-siRNA transfection. *P = 0.0030. miR-122 levels were assessed by northern blot analysis after resolving total RNA on a 20cm × 20cm denaturing polyacrylamide gel (D). The numbers below the lanes indicate the estimated miRNA level compared with the scrambled siRNA (Ctrl)-transfected control, quantified as described in Fig 1C. Levels of GLD-2, NS3, and NS5B proteins were analyzed by immunoblotting. α-tubulin was used as an internal control for loading. (E) R-1 cells treated with increasing concentrations of GLD-2 siRNA were left untreated or transfected with pcDNA3.1-Flag-core (Core) prior to quantification of miR-122 levels as described in (A). *P < 0.01. Where shown, error bars are standard deviations of three independent experiments. *P* values were calculated by unpaired *t*-test.

Having found that HCV infection interferes with miR-122 3′-end tailing in liver tissues, we investigated whether the HCV core protein inhibits this modification. miRNA tailing can be catalyzed by various nucleotidyl transferases including non-canonical poly(A) polymerases and terminal uridylyl transferases. We silenced each of a series of known terminal nucleotidyl transferases using specific small interfering RNAs (siRNAs) and found that miR-122 abundance is specifically regulated by GLD-2, a non-canonical cytoplasmic poly(A) polymerase [29] [also known as terminal uridylyl transferase 2 (TUTase-2)] (Fig 4A). Silencing of GLD-2 expression with increasing doses of siRNA (Fig 4B), which was not associated with a substantial cell viability decrease (Fig 4C), decreased the cellular abundance of three major miR-122 isomers (21-, 22-, and 23-nt isomers) (Fig 4D), resulting in an overall 49% decrease in their miRNA levels and consequent decreases in HCV subgenomic RNA and viral protein (NS3 and NS5B) levels (Fig 4B and 4D). As observed in GLD-2 knock-downed cells, the 23-nt miR-122 isomers as well as the two other major isomers (21-nt and 22-nt species) concurrently decreased both in Huh7 cells transiently expressing the core protein and in HCV-infected cells (S2A and S2B Fig), suggesting that the miR-122 level reduction does not occur in a step-by-step manner in the order of decreasing length of isomers. Furthermore, when the core protein was expressed in GLD-2 knock-downed cells, miR-122 levels decreased further (Fig 4E, see the cells treated with 1 or 5 nM siGLD-2). However, when GLD-2 expression was substantially silenced with higher concentrations of siRNA, this inhibitory effect of core protein was diminished, suggesting that miR-122 level regulation by core protein is a GLD-2-dependent event.

**Fig 5:**
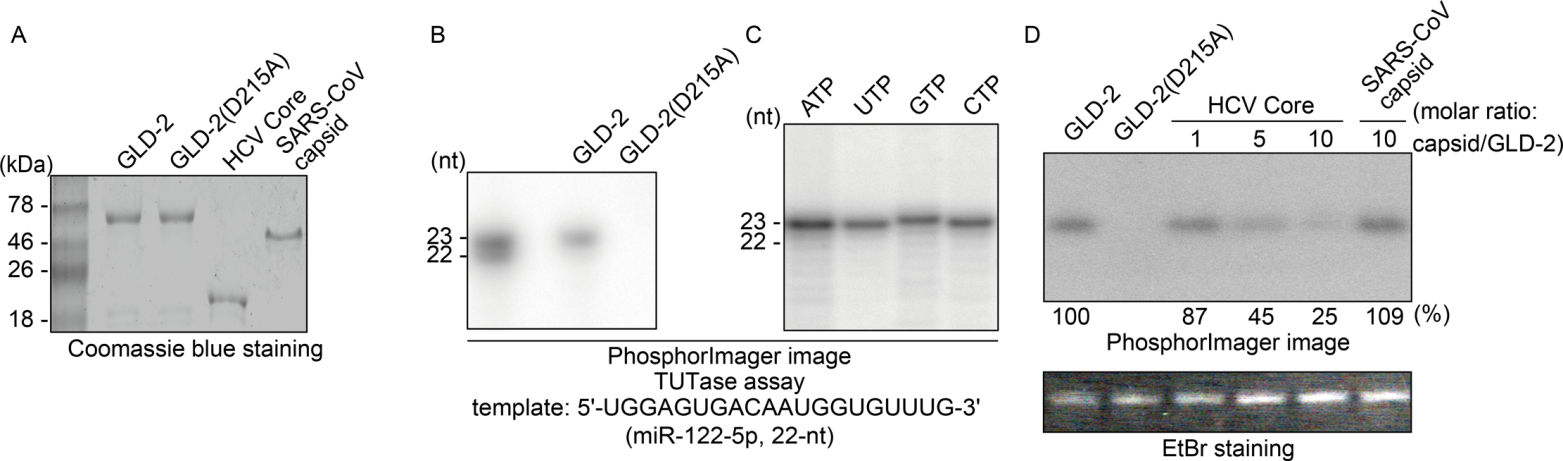
Inhibition of GLD-2 by the HCV core protein. (A) Coomassie blue staining of purified recombinant proteins used in terminal transferase assays. (B and C) A terminal transferase assay with miR-122-5p (22-nt long guide-strand RNA) and GLD-2 or its inactive form GLD-2(D215A) in the presence of [α-^32^P]ATP (B) or each of the indicated radiolabeled ribonucleotides (C). Radiolabeled RNA products were resolved by PAGE on a denaturing polyacrylamide gel (15%) and visualized using a PhosphorImager. As size markers, 5′-^32^P-labeled miR-122 isomers were used. (D) Inhibition of GLD-2 3′ adenylation activity by the HCV core protein. The amount of the HCV core protein or SARS-CoV capsid protein added to the reactions is shown as a molar ratio to GLD-2. The radioactivity present in the bands was quantified using a PhosphorImager and expressed as a percentage compared with the GLD-2 alone control. Shown below the autoradiogram is ethidium bromide (EtBr) staining of the gel showing the amounts of miR-122 template used in the assays.

Because miR-122 abundance was specifically regulated by GLD-2 silencing and the miR-122 isomers’ 3′ end was found to carry four different single ribonucleotides, we assessed the possibility that GLD-2 adds ribonucleotides other than adenylate to the 3′ end of miR-122 and sought to test whether GLD-2 catalytic activity is inhibited by the HCV core protein. We addressed these questions using highly purified recombinant GLD-2 and core proteins (Fig 5A). Initially, we tested the adenylation activity of the purified recombinant GLD-2 with an N-terminal histidine tag and found that wild-type GLD-2, but not its inactive form GLD-2(D215A), monoadenylates miR-122-5p (Fig 5B). This result was unexpected because GLD-2 was originally identified as a non-canonical cytoplasmic poly(A) polymerase that acts on certain mRNAs [29, 30] but was consistent with previous studies illustrating GLD-2-mediated miR-122 3′ adenylation [19, 31]. Importantly, we found that GLD-2 was capable of adding any of the four ribonucleotides to the miR-122 3′ end with almost similar efficiency (Fig 5C). Similarly, GLD-2 added any of four ribonucleotides to the 3′ end of eight other miRNAs (S5A Fig) randomly selected from the top 50 most abundantly expressed miRNAs in human liver, with different efficiencies (S5B Fig). Surprisingly, miR-122-5-p was most efficiently modified by GLD-2. Further analysis of GLD-2 template specificity using ribonucleotide homopolymers (20-nt) revealed that (A)_20_ is the best template of GLD-2 for 3′-end adenylation, uridylation, guanylation, and cytidylation (S5C Fig). These findings suggest that GLD-2 has a certain degree of selectivity on small RNAs for 3′ end modification. Using the miR-122-5p that was most efficiently adenylated by GLD-2, we revealed that the HCV core protein inhibits GLD-2’s 3′-end monoadenylation activity, whereas severe acute respiratory syndrome coronavirus (SARS-CoV) capsid protein had little effect on GLD-2 activity (Fig 5D).

Because GLD-2 silencing lowered miR-122 levels, we assessed whether the HCV core protein expression downregulates GLD-2, but we did not observe a reduction in GLD-2 expression (S6A Fig). Translin, a DNA-binding protein, was reported to bind to miR-122 and increase its stability [32]. Translin mRNA expression level was not altered by the core protein (S6B Fig). These results together suggest that the inhibition of GLD-2-catalyzed 3′ non-templated nucleotide addition by the core protein is responsible for the downregulation of miR-122 following HCV infection.

### Interaction of the HCV core protein with GLD-2 in the cytoplasm is required for the downregulation of miR-122

**Fig 6:**
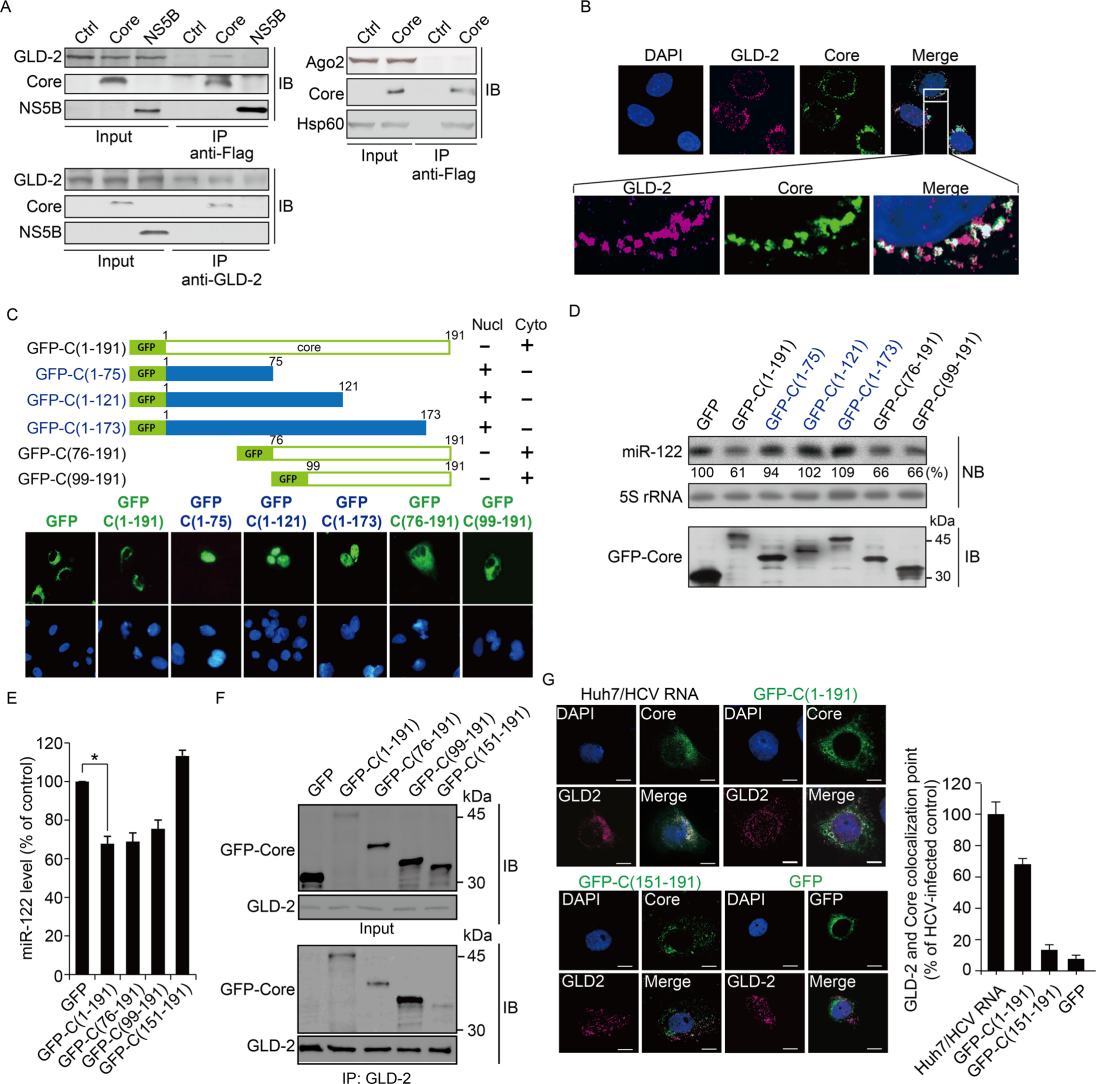
Interaction of the HCV core protein with GLD-2 in the cytoplasm is required for miR-122 expression regulation. (A) Huh7 cells were transfected with an empty vector (Ctrl) or an expression vector encoding Flag-tagged core (Core) or NS5B protein. Lysates were immunoprecipitated with anti-Flag (top) or anti-GLD-2 (bottom) antibody, followed by immunoblotting for the indicated proteins. (B) Indirect double-immunofluorescence staining of the core protein and GLD-2 in Huh7 cells transiently expressing the Flag-tagged core protein. Nuclei were visualized by DAPI staining. (C and D) Subcellular localization of GFP-fused full-length HCV core protein and its truncated derivatives transiently expressed in Huh7 cells for 48 h was analyzed by immunofluorescence microscopy (C). Nucl, nucleus; Cyto, cytoplasm. (D) represents the levels of miR-122 and GFP-fused core proteins measured by northern blot (NB) analysis and immunoblotting (IB). The numbers below the northern blot indicate the miR-122 level compared with the GFP-transfected control, quantified as described in Fig 1C. Total cell lysates resolved by SDS-PAGE were analyzed by immunoblotting using anti-GFP antibody (bottom). (E) RT-PCR quantification of miR-122 levels in Huh7 cells transiently expressing GFP or the indicated GFP-fused core proteins. (F) Analysis of interaction between GLD-2 and the indicated GFP-fused core proteins by co-immunoprecipitation experiments. (G) Quantitative analysis of colocalization between GLD-2 and the indicated GFP-fused core proteins in Huh7 cells. Huh7/HCV RNA, Huh7 cells transfected with HCV (JFH-1) RNA. Scale bar, 10 μm.

To verify the interaction between the core protein and GLD-2, we performed coimmunoprecipitation experiments using cell lysates from the Huh7 cells in which Flag-tagged HCV core protein or NS5B protein was ectopically expressed. Fig 6A demonstrates the ability of the core protein to interact with endogenous GLD-2. HCV core protein did not interact with Ago2, whereas its association with Hsp60 [33] could be confirmed. This result suggests that miR-122 level regulation was not mediated through Ago2-core protein interactions. Coimmunofluorescence staining showed that the HCV core protein, which predominantly resided on the perinuclear region, partially colocalized with GLD-2, which was previously identified to be located in both the nucleus and cytoplasm [28], when they are present in the cytoplasm (Fig 6B).

In an additional experiment using various GFP-tagged core proteins (Fig 6C, top panel), we found that the full-length core protein GFP-C(1–191) and its truncation mutants GFP-C(76–191) and GFP-C(99–191), which localized in the cytoplasm due to the presence of the C-terminal region spanning amino acids 174–191, downregulate miR-122 expression. In contrast, other truncation mutants [GFP-C(1–75), GFP-C(1–121), and GFP-C(1–173)], which translocated to the nucleus, failed to decrease miR-122 levels (Fig 6C and 6D), indicating that GLD-2 inhibition-mediated miR-122 expression regulation by the core protein is a cytoplasmic process. These results also indicate that the RNA-binding activity of the core protein is not involved in this regulation because GFP-C(76–191) and GFP-C(99–191), lacking the known RNA-binding domain (amino acids 1–75) [34], did also reduce miR-122 levels. GFP-C(151–191), in which 52 amino acids was further deleted from GFP-C(99–191) and thus lacks the most of the domain 2 of HCV core protein known to be required for its lipid droplet association [35], failed to decrease miR-122 level (Fig 6E). This mutant showed a weaker affinity to GLD-2 as revealed by co-immunoprecipitation experiments (Fig 6F). More importantly, this deletion mutant was barely colocalized with GLD-2 in the cytoplasm, whereas GFP-C(1–191) and core protein expressed in Huh7 cells by transfection of HCV RNA colocalzied with GLD-2 in the perinuclear regions and lipid droplet-like structures (Fig 6G). HCV RNA (JFH-1 *in vitro* transcripts)-transfected Huh7 cells showed enhanced colocalization of the core protein with GLD-2 compared with the cells expressing the GFP-fused core protein alone, suggesting that core protein-GLD-2 interaction in cells might be affected by viral protein expression and/or viral genome replication.

### 3′-End nucleotide addition to miR-122 promotes its activity and stability

**Fig 7:**
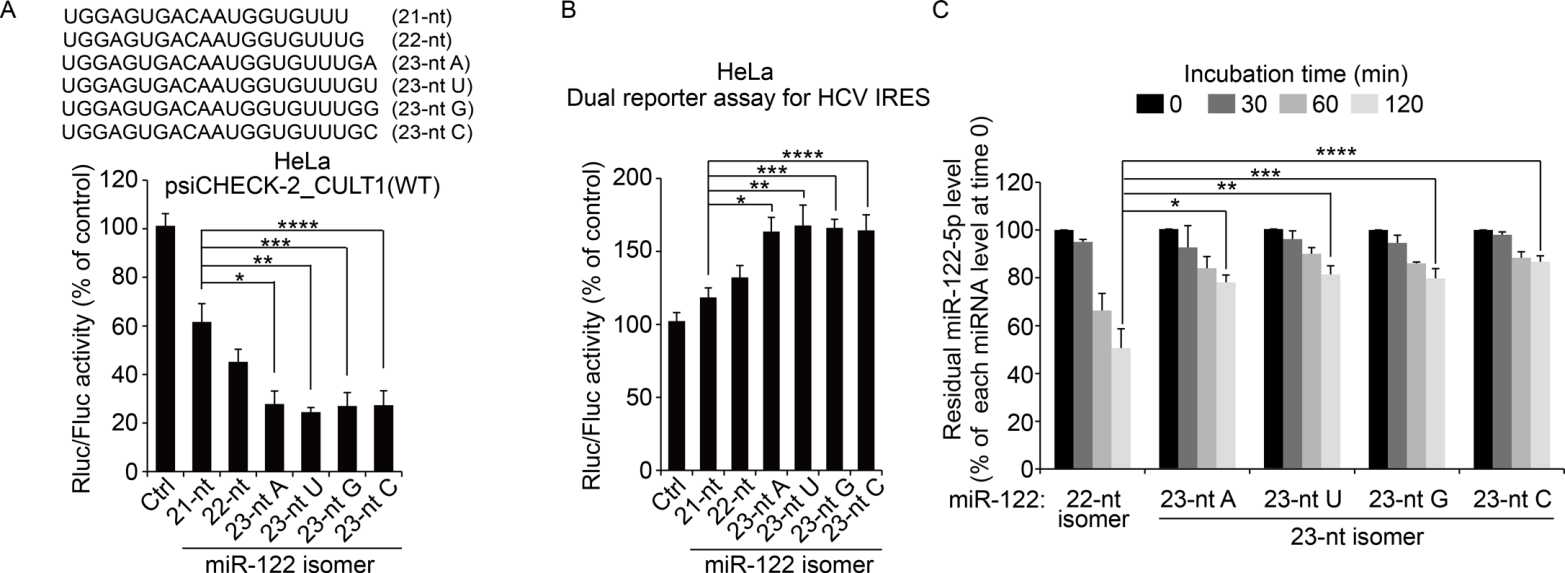
Analysis of the activity and stability of miR-122 isomers. (A) A reporter assay was performed 48 h after the transfection of duplex forms of the indicated miR-122 isomers or scrambled siRNA (Ctrl) and the reporter plasmid psiCHECK-2_CULT1(WT) harboring a miR-122 target. Shown is the normalized luciferase activity (Rluc/Fluc) compared with the scrambled siRNA-transfected control. *P = 0.02; **P = 0.017; ***P = 0.034; and ****P = 0.037. (B) HeLa cells were co-transfected with duplex forms of the indicated miR-122 isomers and a dual luciferase reporter allowing HCV IRES-mediated Rluc translation. After 48 h, transfected cells were harvested, and the relative normalized luciferase activity (Fluc/Rluc) was measured as described in Fig 2E. *P = 0.002; **P = 0.0017; ***P = 0.0034; and ****P = 0.0037. (C) Improved stability of 23-nt miR-122 isomers compared with the most abundant, prototype 22-nt miR-122. Residual miR-122 guide strand abundance measured by northern blot analysis after incubation of individual duplex miR-122 isomers with HeLa cell extracts for the indicated periods. *P = 0.0012; **P = 0.0018; ***P = 0.0012; and ****P = 0.0013. In all panels, error bars are standard deviations of three independent experiments. *P* values were calculated using an unpaired *t*-test.

We investigated whether miR-122 3′-end modification with four different ribonucleotides has any impact on miRNA activity and stability. In HeLa cells, which express an undetectable amount of miR-122 (S7 Fig), all of the 23-nt miR-122 isomers (pre-annealed imperfect duplex miRNAs modified with a single nucleotide addition added to their 3′ ends) significantly increased the reporter-suppressing activity compared with the findings using the 21-nt isomer in a reporter assay with the psiCHEK-2_CULT1(WT) plasmid (Fig 7A). Similarly, these miR-122 isomers were more effective in enhancing HCV IRES-mediated translation compared with the shorter forms of miR-122, the 21- and 22-nt isomers (Fig 7B). We found that the single nucleotide-extended miR-122 isomers displayed improved stability compared with the 22-nt isomer when their cellular stability was assessed by northern blot analysis for the residual amounts of miR-122-5p following the incubation of individual duplex miRNAs in HeLa cell extracts (Fig 7C). These results demonstrate that miR-122 isomers modified by GLD-2-mediated 3′ non-templated addition display increased miRNA activity and stability, implying that GLD-2 inhibition by the HCV core protein promotes miR-122 destabilization to dysregulate its functions in the liver and in the HCV life cycle.

## Discussion

In this study, we examined the mechanism by which the cellular abundance of miR-122 is downregulated by HCV. Diverse regulatory mechanisms, which include the transcription regulation and post-transcriptional turn-over control of mature miRNA, might explain the differential expression of miRNAs in HCV-infected cells. We demonstrated that the HCV core protein inhibits GLD-2 and thereby promotes miR-122 destabilization, which leads to the downregulation of HCV RNA abundance.

Inhibition of GLD-2 by the HCV core protein affects multiple miRNAs in addition to miR-122 by decreasing the proportion of mono-adenylated isomers. Deep sequencing analysis of small RNAs from liver biopsies revealed that among the top 50 most abundantly expressed miRNAs in liver tissues, only six miRNAs exhibited a decrease in their 3′-end monoadenylated isomer levels upon HCV infection. The cellular abundance of these miRNAs was diverse (S3A Fig), suggesting that the selection of the miRNA template does not appear to be determined by the abundance of miRNAs alone. Instead, there might be a certain template specificity for GLD-2. However, the six miRNAs did not have any conserved sequences (S4A Fig).

GLD-2 has no known RNA-binding motif [29, 30]. Thus, it is recruited by the phosphorylated CPEB/CPSF complex to the 3′-UTR of target mRNAs for cytoplasmic poly (A) addition [36]. Similarly, in a cellular context, helper protein might also govern GLD-2 specificity toward miR-122. Notably, the miRNA species, for which the adenylated isomer levels were decreased by HCV infection, were not perfectly matched to the GLD-2 depletion-sensitive (in terms of stability) miRNAs in human fibroblasts [19]. Thus, the type of miRNAs post-transcriptionally modified by GLD-2 might be determined by cell-type specific cofactors used by GLD-2 to recognize a specific set of miRNAs. Nevertheless, GLD-2 appears to a higher affinity for miR-122-5p preferentially for mono-ribonucleotide addition to its 3′-end compared with either other liver resident miRNAs we tested (S5B Fig). Surprisingly, GLD-2 displayed a much higher terminal transferase activity on 20-mer homopolymer (A) than other homopolymeric RNA templates (S5C Fig). These results suggested that miRNA sequence and/or structure might also important determinants for GLD-2 template selectivity.

Given that miR-122 is a specific template of GLD-2, one important question is whether the non-templated addition of all four different nucleotides to miR-122 is catalyzed by GLD-2 alone. An early study claimed that *C*. *elegans* GLD-2 from reticulocyte lysates programmed with a plasmid encoding GLD-2 exhibited a strict ATP dependency on a C_35_A_10_ substrate [29]. GLD-2 monoadenlyation activity on miR-122 was also previously demonstrated with an immunoprecipitated mouse GLD-2 [19, 31] and a recombinant GDL-2 fused to the C-terminal end of GST [19]. Interestingly, the GST-fused GLD-2 used in the latter study displayed a weak preference for ATP over UTP on miR-122. Our terminal nucleotidyl transferase assays conducted using His-tagged recombinant GLD-2 revealed that any of the four ribonucleotides can be added to the 3′-end of miR-122 without preference for ATP (Fig 5C), raising the possibility that the non-templated additions of all four different nucleotides to the miR-122 3′ end might be catalyzed by GLD-2. Supporting this hypothesis, we detected the 23-nt miR-122 isomers modified by the non-templated addition of a single A, G, or C residue in five different liver biopsies analyzed. The ability of GLD-2 to use UTP and ATP as a substrate was also observed in the 24-nt isomers in which the U-tailed 23-nt isomer (it can be derived by the non-templated addition or aberrant processing of pre-miR-122) was further extended by the addition of a single uridylate or adenylate residue. Owing to the high abundance of miR-122 in the liver, the analysis of our small RNA sequencing datasets enabled us to identify previously uncovered 24-nt miR-122 species that carry a dinucleotide (AA and AU; all represent non-templated nucleotides that always start with A) or a 25-nt species bearing a triple nucleotide sequence (UUU; underlined sequences represent non-templated nucleotides). In these miR-122 species, the non-templated addition of uridylate residues was repeatedly observed. These results further suggest that GLD-2 may have an intrinsic capability of using diverse nucleotides as substrates. If consistent with the *in vitro* findings for GLD-2, the inhibition of GLD-2 by the HCV core protein would lower the proportion of miR-122 isomers bearing any single non-templated nucleotides at their 3′ ends. Indeed, the read frequency of all of the abovementioned miR-122 species was substantially decreased in liver biopsies from patients with HCV (S3 Table). A study illustrated that the Zcchc11-dependent uridylation of miR-26a and 26b attenuates miRNA-target repression [37]. However, uridylation of miR-122 enhanced its stability and miRNA activity, highlighting the importance of the fine regulation of miRNA 3′-end modification for executing the optimal functions of individual miRNAs. Finally, it should be stated that there was a substantial decrease in the proportion of the single U-tailed abundantly present 23-nt miR-122 isomer (aberrantly processed or terminally modified one), suggesting that pre-miR-122 processing in its single-stranded loop region might also be influenced by HCV infection (30%–33% of 22-nt canonical miR-122 in normal liver versus 14%–18% in patient liver biopsies). This interesting issue is currently under further investigation.

In addition to its role as a structural protein, the HCV core protein is also known to be involved in the development of hepatocellular carcinoma [38]. The tumor suppressor activity of miR-122 is well characterized in cells and miR-122 knockout mice [12–15]. Thus, our results imply that the HCV core protein may contribute to liver cancer development by decreasing miR-122 levels. Decreased miR-122 expression resulting from core protein expression during the course of chronic HCV infection may in part explain the oncogenic phenotype previously observed in cell culture and transgenic mice expressing the core protein [38]. Furthermore, the core protein-expressing transgenic mice and miR-122 knockout mice both are characterized by hepatic inflammation and fibrosis, followed by the development of hepatocellular carcinoma with age, further supporting the role of the core protein in liver diseases through its ability to promote miR-122 destabilization by inhibiting GLD-2. Studies have suggested that let-7i-5p and miR-29a-3p act as tumor suppressors because of their ability to repress oncogenes [39, 40]. It remains to be investigated whether the functions of these anti-cancer miRNAs are regulated by alteration in their 3′-end modification as observed in liver biopsies from patients with HCV (S4 Fig). If this is confirmed, GLD-2 inhibition by the core protein can be one of the mechanisms accelerating tumor development by chronic HCV infection through a previously uncovered pathway. This hypothesis is supported by the finding that GLD-2 is downregulated in several cancers, as revealed by a gene expression meta-analysis [41], implying that the loss of GLD-2 could be involved in carcinogenesis.

Generally, cytoplasmic polyadenylation by GLD-2 with the help of GLD-3, as illustrated in a recent structural analysis of these proteins [30], is required for the stability and translational activity of mRNAs [30, 42]. So, what might be the consequence of GLD-2 inhibition by the core protein? We can speculate that cytoplasmic poly (A) addition to some host mRNAs 3′-UTR can be inhibited by the HCV core protein. Inhibition of host translation by virus infection is mediated by the inactivation of translation initiation factor 2 through its phosphorylation by PKR, a type I interferon inducible gene [43]. Because many viruses, including HCV, suppress or abrogate the type I interferon signaling pathway and PKR activation by diverse mechanisms [44], whereas subgroups of host mRNAs are yet subjected to down-regulation, it is tempting to speculate that GLD-2 inhibition by the HCV core protein affects host mRNA translation. It remains to be characterized what host mRNAs are subjected to this regulation to understand the impact of the chronic inhibition of GLD-2 in liver pathophysiology.

In summary, our results provide a novel mechanism by which the HCV core protein controls viral replication levels through the downregulation of miR-122. The beneficial effect of miR-122 in HCV replication and translation can be repressed by the accumulation of the core protein, which determines the degree to which the host supports viral replication and thus plays a role in establishing persistent infection. Suppressing miR-122 levels in addition to sequestering of miR-122 by the HCV genome as reported recently [45] might be viral strategies to control viral titers. These miR-122 suppressing effect would also contribute in promoting liver diseases and cancer development.

## Materials and Methods

### Plasmids, antibodies, and reagents

The pcDNA3.1-Flag-core plasmid expressing the Flag epitope-tagged full-length HCV core protein was described previously [46]. The pcDNA3.1-Flag-NS5B expressing the Flag epitope-tagged full-length HCV NS5B was previously constructed [47]. The pEGFP-C(1–191), pEGFP-C(1–75), pEGFP-C(1–121), pEGFP-C(1–173), pEGFP-C(76–191), pEGFP-C(99–191) [48], and pEGFP-C(151–191), in which the numbers in parentheses indicate the amino acid positions in the core protein, were used for the expression of the enhanced green fluorescence protein (EGFP)-fused HCV core protein and its deletion derivatives. psiCHECK-2_CULT1(WT) and psiCHECK-2_CULT1(MT) were kindly provided by Liang-Hu Qu (Sun Yat-Sen University, China). These dual luciferase reporters contain the 3′-UTR fragment of cut-like homeobox 1 (i.e., CULT1), a transcriptional repressor of genes specifying terminal differentiation in hepatocytes, carrying either the miR-122 target sequence or its mismatched target sequence (Fig 2A) [23]. The pTrcHisB-GLD-2 plasmid was constructed by inserting GLD-2 cDNA amplified by RT-PCR using forward (5′-CCGGCTAGCATGTTCCAAACTCAATTTTGGG-3′) and reverse (5′-CGAAGCTTTTATCTTTTCAGGACAGCAGCTC-3′) primers into the pTrcHisB vector (Invitrogen, Carlsbad, CA, USA) via the *Nhe*I and *Hin*dIII sites. The pTrcHisB-GLD-2(D215A) was generated by site-directed mutagenesis to express an inactive GLD-2(D215A) carrying an Ala substitution for Asp215 at the catalytic active site of GLD-2 [19]. The bicistronic vector pDual-IRES, which expresses a cap-dependent Rluc reporter and an HCV IRES-dependent Fluc reporter was described previously [49]. The JFH1 plasmid [50] was used to produce infectious HCV particles. Antibodies were obtained as follows: rabbit polyclonal anti-GFP antibody from Santa Cruz Biotechnology (Santa Cruz, CA, USA), rabbit polyclonal anti-GLD-2 antibody, rabbit polyclonal anti-NS5A antibody, mouse monoclonal anti-NS5B antibody (clone 10D6), and mouse monoclonal anti-core protein antibody (clone C7-50) from Abcam (Cambridge, MA, USA), rabbit polyclonal anti-Ago2 antibody and mouse monoclonal anti-Hsp60 antibody (clone EPR4211) from Cell Signaling Technology (Danvers, MA, USA), and mouse monoclonal anti-α-tubulin antibody (clone DM1A) from Calbiochem (La Jolla, CA, USA). SARS-CoV capsid protein and the HCV core protein, which were expressed in *Escherichia coli* as N-terminal His-tagged full-length forms, were prepared as previously described [46, 51]. miRNAs were obtained from ST Pharm (Seoul, Korea). The sequences of miRNAs used in this study are shown in S5A Fig.

### Cell culture and HCV infection

Huh7 human hepatocellular carcinoma cells (ATCC, Manassas, VA, USA) were grown in Dulbecco’s modified Eagle’s medium supplemented with 10% fetal bovine serum, 2 mM l-glutamine, 100 U/ml penicillin, 100 μg/ml streptomycin, and 0.1 mM nonessential amino acid under standard culture conditions (5% CO_2_, 37°C). The Huh7 stable cell lines Huh7/core and Huh7/NS5B, which express Flag-tagged core and NS5B proteins, respectively, were obtained previously by transfecting pcDNA3.1-Flag-core [46] and pcDNA3.1-Flag-NS5B [47]. The Huh7-derived cell line R-1 harboring a genotype 1b HCV subgenomic replicon was previously described [52]. Primary hepatocytes from BALB/c mice were isolated using a two-step perfusion method and grown on collagen-coated culture plates as previously described [53]. Huh7 cells were infected with HCV particles, which were recovered from Huh7 cells transfected with full-length HCV (genotype 2a, JFH1 clone) RNA at a multiplicity of infection (MOI) of ~0.25, as previously described [54].

### Small RNA sequencing

Total RNA was extracted from human liver biopsies (N-1, N-2, HCV-1, HCV-2, and HCV-3) using Trizol reagent (Invitrogen). Details on the human liver biopsy specimens used in this study are given in the Supplemental Information S3 Table. The small RNA fraction was enriched from total RNA extracted from human liver biopsies (N-1, N-2, HCV-1, HCV-2, and HCV-3) using a *mir*Vana miRNA isolation kit (Ambion, Austin, TX, USA). Similarly, small RNA fraction was prepared from Huh7 cells transiently expressing HCV core protein or infected with HCV. The cDNA library for small RNA was prepared for Illumina sequencing using a Truseq Small RNA Sample Preparation kit (Illumina, San Diego, CA, USA), according to the manufacturer’s protocol. The amplified PCR products were analyzed by electrophoresis on an agarose gel, and the DNA band of an appropriate size was then excised from the gel. The cDNA amplicons were analyzed on a Bioanalyzer High Sensitivity DNA chip (Agilent, Santa Clara, CA, USA). The deep sequencing libraries were sequenced on an Illumina Genome Analyzer GA II (Illumina) using the 54-bp single read protocol. Sequencing data were analyzed using the bowtie-1.0.1 program.

### Bioinformatic analysis of small RNA libraries

Solexa sequence reads were subjected to adapter removal using the FASTX-toolkit (http://hannonlab.cshl.edu/fastx_toolkit/). After removal of redundant reads, the reads (16–30 nt) were mapped to the human reference genome hg19 in the UCSC Genome Browser Database [55] using the Bowtie program (version 1.0.1) with the following options: -v 2 -m 10 –best—strata. The reads that could be aligned to the human genome were moved to the “mapped” dataset, and suppressed reads were discarded to prevent multiple mapping. After mapping, only unique reads of “mapped” dataset were annotated using the miRBase database (release 19) (http://www.mirbase.org) for miRNA [56]; the Rfam database (release 11) (http://rfam.xfam.org) for rRNA, various noncoding small RNAs, and repeat sequences [57]; and the genomic tRNA database (http://gtrnadb.ucsc.edu/Hsapi19/) [58]. Individual mapped sequence reads were compiled into a set of unique sequences with the read counts for each sequence reflecting the relative abundance. The unique sequence read counts were normalized to the total read counts of the “mapped” dataset in millions to give reads per million.

### qRT-PCR

The HCV genome copy number was estimated by real-time qRT-PCR using an HCV 5′-UTR-specific TaqMan probe as previously described [59]. Mature miRNA (miR-122 and miR-221) quantification was performed using TaqMan miRNA assays (Applied Biosystems, Foster, CA, USA), according to the manufacturer’s instruction. Glyceraldehyde-3-phosphate dehydrogenase (GAPDH), miR-122 precursor, and GLD-2 levels were estimated by qRT-PCR using a SYBR Green assay kit (Takara, Kyoto, Japan). The primers used for qRT-PCR were as follows: GAPDH (forward primer, 5′-GAAGGTGAAGGTCGGAGTC-3′; reverse primer, 5′-GAAGATGGTGATGGGATTTC-3′), miR-122 precursors (pri-miR-122 and pre-miR-122: forward primer, 5′-GCCTAGCAGTAGCTATTTAGTGTG-3′; reverse primer, 5′-CCTTAGCAGAGCTGTGGAGT-3′), and GLD-2 (forward primer, 5′-GTCTAGAGCTGTGTCATTACAGCA-3′; reverse primer, 5′- TCGCTTAATCTCTTCCTTCCTCG-3′). U6 snRNA levels were determined as described previously [60]. Gene expression levels, normalized to GAPDH unless otherwise specified, were determined using the ΔΔCt method as previously described [61]. Data are shown as the mean ± SD of three experiments, each involving triplicate PCR assays.

### Northern blot analysis

Total RNA (20 μg) isolated from Huh7 cells using Trizol reagent was resolved by electrophoresis on a 15% denaturing polyacrylamide gel, transferred onto a positively charged nylon membrane (Roche Diagnostics, Mannheim, Germany), and fixed to the membrane by UV crosslinking. The membrane was pre-hybridized for 30 min and then hybridized with a 5′-radiolabeled antisense probe overnight. The probe was generated using [γ-^32^P] ATP and T4 polynucleotide kinase (Takara). After washing, the blot was analyzed with a PhosphorImager. Probe sequences were as follows: 5′-ACAAACACCATTGTCACACTCCA-3′ (miR-122-5p), 5′-GAAACCCAGCAGACAATGTAGC-3′ (miR-221), and 5′-CCTGCTTAGCTTCCGAGATCA-3′ (5S rRNA).

### Western blotting and immunoprecipitation

Cell lysates were prepared in a lysis buffer (1% Triton X-100, 100 mM Tris-HCl, pH 8.0, 150 mM NaCl, 10 mM NaF, 1 mM Na_3_VO_4_, and 17.5 mM β-glycerophosphate) supplemented with a protease inhibitor cocktail (Roche Diagnostics). Flag-tagged HCV core protein and NS5B were immunoprecipitated using anti-Flag-M2 affinity resin (Sigma-Aldrich, San Jose, CA, USA) from cell lysates, following incubation for 1 h on a rotator at 4°C in the presence of 2 μg/ml RNaseA (Sigma-Aldrich). Immunoprecipitates or lysates (30 μg) were separated by sodium dodecyl sulfate-polyacrylamide gel electrophoresis (SDS-PAGE) and were blotted onto nitrocellulose membranes. For detection of HCV HCVcore protein in chimeric mice, liver tissues were homogenized, mixed with an equal volume of a standard RIPA lysis buffer (50 mM Tris-HCl, pH 7.5, 150 mM NaCl, 1% NP40, 0.1% SDS, 0.5% sodium deoxycholate) supplemented with a protease inhibitor cocktail, and cleared by centrifugation. The clear lysates (100 μg) were analyzed by immuoblotting. Membranes were analyzed using various primary antibodies and appropriate horseradish peroxidase-conjugated secondary antibodies. Immunoblots were developed using the ECL detection kit (GE Healthcare Life Sciences, Piscataway, NJ, USA).

### Luciferase assay

Huh7 cells seeded at 3 × 10^5^ cells/well in a 6-well plate were grown overnight and transfected with a dual luciferase reporter plasmid (1 μg) using Fugene HD (Promega, Madison, WI, USA), according to the manufacturer’s protocol. Luciferase assays were performed 48 h after transfection using the Dual-Glo luciferase assay system (Promega).

### Cholesterol analysis

Total cholesterol was extracted from cells as described previously [62]. Cholesterol content was determined using the Amplex Red cholesterol assay kit (Molecular Probes, Eugene, OR, USA).

### Design of siRNAs

siRNAs targeting GLD-2 and other nucleotidyl transferases were designed according to the GLD-2 mRNA sequence (GenBank NM 001114394) and previous studies [63], respectively, and were obtained from ST Pharm (Seoul, Korea). Their antisense sequences (all siRNAs have a UU-3′ overhang) were as follows: 5′-CGAGCACAUUCACUAACAA-3′ (TUTase-1; also known as mtPAP, PAPD1, and Hs4), 5′-CUGAACAAUUGCCUUAAGU-3′ (GLD-2; also known as TUTase-2 and PAPD4), 5′-GGACGACACUUCAAUUAUU-3′ (TUTase-3; also known as PAPD5 and TRF4-2), 5′-UGAUAGUGCUUCAGGAAUU-3′ (TUTase-4; also known as ZCCHC11, Hs3, and PAPD3), 5′-CUACGGUACCAAUAAUAAA-3′ (TUTase-5; also known as PAPD7 and TRF4-1), 5′-GCAGCCAAUUACUGCCGAA-3′ (TUTase-6; also known as U6 TUTase, PAPD2, Hs5, and TUT1), and 5′-GAAAAGAGGCACAAGAAAA-3′ (TUTase-7; also known as ZCCHC6 and PAPD6). siRNAs were transfected into cells using Lipofectamine RNAiMAX (Invitrogen), according to the manufacturer’s instructions

### Expression and purification of recombinant GLD-2

*E*. *coli* BL21 transformed with pTrcHisB-GLD-2 were cultured in LB medium containing 100 μg/ml ampicillin. Cells were cultured at 37°C to 0.8 OD at 600 nm, and protein expression was induced at 25°C by the addition of 1 mM isopropyl-β-d-thiogalactopyranoside for 12 h. Cell pellets from a 2-l culture were washed once with phosphate-buffered saline (PBS) and resuspended in 40 ml of binding buffer (50 mM Tris-HCl, pH 8.0, 300 mM NaCl, 10 mM imidazole, 10 mM β-mercaptoethanol, 10% glycerol, 1% Nonidet P-40). Cells were sonicated on ice and centrifuged at 35,000 × *g* for 15 min. After centrifugation, the supernatant was bound to Ni-nitrilotriacetic acid agarose resin (Qiagen, Hilden, Germany) pre-equilibrated with the binding buffer. Bound proteins were eluted with the binding buffer containing imidazole (50–500 mM). Fractions containing GLD-2 were then dialyzed against buffer A [50 mM Tris-HCl, pH 8.0, 1 mM dithiothreitol (DTT), 50 mM NaCl, 5 mM MgCl_2_, and 10% glycerol], and aliquots were stored at −80°C.

### GLD-2 nucleotidyl transferase assay

Terminal nucleotidyl transferase reactions were set up in a total volume of 25 μl containing 50 mM Tris-HCl (pH 7.5), 50 mM NaCl, 5 mM MgCl_2_, 1 mM DTT, 20 U of RNase inhibitor (Promega), 10 μCi of [α-^32^P] rNTP (3000 Ci/mmol, Amersham Pharmacia Biotech), 250 ng (33.7 pmol) of miRNA or 30 pmol ribonucleotide homopolymer (20-nt), and 3.75 pmol of purified GLD-2. After 30 min of incubation at 37°C, reactions were stopped by adding 60 μl of an acid phenol emulsion [phenol:chloroform:10% SDS:0.5 M EDTA (1:1:0.2:0.4)] and 20 μg of glycogen. RNA products were precipitated with 2.5 volumes of cold 5 M ammonium acetate-isopropanol (1:5) and washed with 80% cold ethanol. After heat denaturation, the RNA samples were subjected to electrophoresis on an 8 M urea–15% polyacrylamide gel and were visualized by autoradiography. Densitometric quantification of radioactivity was performed using a Fuji BAS-2500 PhosphorImager.

### Immunocytochemistry

Huh7/core or HCV-infected Huh7 cells were cultured in four chamber slides to 50% confluency. After 48 h, cells were fixed with 4% paraformaldehyde in methanol for 15 min at room temperature, washed 3 times with cold PBS, and then permeabilized with PBS containing 0.2% Triton X-100 for 30 min at room temperature. After washing three times with PBS, the cells were treated with a blocking solution (3% horse serum in PBS) for 30 min at room temperature. Cells were further incubated with an anti-GLD2 antibody overnight at 4°C and then washed three times with PBS. Cells were further incubated with an Alex Flour 647-conjugated anti-rabbit IgG antibody (Invitrogen) and a FITC-conjugated mouse monoclonal anti-core antibody (clone IE5, Abcam) for 2 h at room temperature and then washed three times with PBS. Nuclei were visualized by staining with 1 μM 4′, 6′-diamidino-2-phenylindole (DAPI) in PBS. Confocal images were collected on an LSM 510 META confocal laser-scanning microscope (Carl Zeiss, Oberkochen, Germany). Colocalization of GLD-2 with HCV core protein was determined by using the colocalization plugin module in the NIH ImageJ/Fiji software (v. 1.50).

Immunostaining for HCV core protein in mouse liver tissues was performed using an anti-core antibody (C7-50) as described previously [64].

### miRNA stability test

Duplex miRNAs (10 pmol) were incubated with HeLa cell lysate (40 μg) in a 110-μl reaction buffer (50 mM Tris-HCl, pH 8.0, 150 mM NaCl, 0.1% NP-40, and 10% glycerol) supplemented with complete EDTA-free 1× protease inhibitor cocktail (Roche Diagnostics) for 0, 30, 60, and 120 min. RNA was extracted from a 25-μl aliquot of the reaction mixture using Trizol LS reagent (Invitrogen) for northern blot analysis.

### Cell viability

Viability of R-1 cells transiently expressing the Flag-tagged core protein was measured using MTS [3-(4,5-dimethylthiazol-2-yl)-2,5-diphenyltetrazolium bromide] reagent as previously described [64].

### Ethics statement

Liver biopsy samples from two healthy volunteers and three patients with HCV were obtained from the National Biobank of Korea, and written informed consent was obtained from all subjects (Pusan National University Hospital, Busan Korea, IRB Approval No: 2011–3). The study protocol was approved by the institutional review board of Korea Advanced Institute of Science and Technology (IRB-14-040). All animal experiments were performed by certified personnel in an approved animal facility of the Yonsei Univeristy, in accordance with the Korean Food and Drug Administration guidelines. BALB/c male mice (6 weeks old; LaonBio Inc, Korea) were used to isolate primary hepatocytes. This study was approved by the Institutional Animal Care and Use Committee (IACUC) of the Yonsei University (Permit No: IACUC-A-201408-274-01). Liver tissues from HCV (genotype 1b patient serum)-infected SCID chimeric mice (uPA^+/+^SCID^+/+^) in which mouse liver was repopulated with human hepatocytes were obtained from Phoenix Bio (Hiroshima, Japan). The experimental protocols for the chimeric mice were approved by the ethics board of the Hiroshima Prefectural Institute of Industrial Science and Technology, Hiroshima, Japan.

### Statistical analysis

Statistical analyses were performed using GraphPad Prism 6.01 (GraphPad Prism Software Inc., La Jolla, CA, USA). Results are presented as the mean ± SD from at least three independent experiments, unless otherwise stated. The *p*-value was calculated using a one-tailed unpaired Student’s *t*-test. *P* values < 0.05 were considered statistically significant.

## Supporting Information

S1 Fig Analysis of core protein and viral RNA titer in HCV-infetced cells. (A) Immunostaining of HCV core protein in HCV-infected Huh7 cells. DAPI, nuclear staining; scale bar, 20 μm. (B) At the indicated time points, miR-122 and HCV core protein levels in HCV-infected Huh7 cells were assessed by RT-PCR and immunoblotting, respectively. (C and D) Analysis of HCV core protein and genome titer in the HCV-infected SCID mice, PXB202 carrying the chimeric liver repopulated with human hepatocytes, by immunostaining (C) and real-time qRT-PCR (D), respectively. The serum HCV titer in PXB202 determined by qRT-PCR was 3 × 10^7^/ml. Scale bar, 100 μm.
(TIF)

S2 Fig Northern blot analysis of miR-122 isomers in Huh7 cells and primary hepatocytes. (A) Total RNA from Huh7 cells transfected with pcDNA3.1 (Ctrl) or pcDNA3.1-Flag-core plasmid (Core) were resolved on a 20cm × 20cm denaturing polyacrylamide gel and subjected to northern blotting for miR-122. (B) Huh7 cells infected with HCV at an MOI of 0.25 were analyzed 2 days after infection as described in (A). (C) Detection of miR-122 isomers in primary hepatocytes isolated from mice (#1 and #2).
(TIF)

S3 Fig 3′-Terminal modification by a single nucleotide addition occurs on all of the top 50 most abundant miRNAs present in the liver, with uridylation and adenylation being two major modification events. Shown are the percentages of the read count of 3′-terminally single nucleotide-tailed isomers compared with the total read count of each miRNA’s major isomers, which include an isomer with a 3′-end 1-nt deletion, prototype miRNAs, and miRNAs bearing a 3′-terminal single nucleotide tail. N-1 and N-2 denote liver biopsies from healthy controls, and HCV-1 to HCV-3 are liver biopsies from patients with HCV. The numbers above or below the bars indicate the estimated mean value of the proportion for each isomer. miRNA isomers are grouped according to the tail sequence at the 3′ end of individual isomers, which can be either derived from a precursor by aberrant processing and/or by non-templated addition (red symbols) or solely by non-templated addition (black symbols). Via miRNA isomer profile analysis for the top 50 most abundantly expressed miRNAs in liver biopsies, we discovered that these 50 miRNAs, including miR-122, are modified via a non-templated or templated 3′ addition of any of four ribonucleotide residues. The analyzed miRNAs frequently contained a single adenylate or uridylate residue rather than guanylate or cytidylate residues. The average frequencies of mono-U, mono-A, mono-G, and mono-C addition were 12.97%, 9.83%, 2.88%, and 4.07% (when accounting for both templated and non-templated additions), respectively, in the normal liver tissue (N-1). The ratios for mono-G and mono-C additions for these miRNAs were relatively low when considering only non-templated addition. Similar profiles were also observed with another normal liver biopsy N-2 and liver biopsy samples from patients with HCV. These results reveal that miRNA 3′-end modification occurs frequently in liver-resident miRNAs with non-templated monoadenylation and monouridylation being two major modification processes.
(TIF)

S4 Fig HCV infection affects 3′-terminal modification of specific miRNAs in the liver. Fold change in the proportion of 3′-end mono-A (A), mono-U (B), mono-G (C), and mono-C (D)-tailed miRNA isomers in liver samples from patients with HCV (HCV-1 to HCV-3, red symbols) compared with the healthy controls (N-1 and N-2, black symbols). Shown are the values for the top 50 most abundant miRNAs present in liver biopsies. miRNAs on the x-axis (starting from miR-122-5p) are in the order of their abundance in liver biopsies. The miRNAs highlighted in red on the x-axis represent those exhibiting >25% decreases in the proportion of the indicated 3′-tailed miRNA isomers in the patient liver biopsies. In (B), the miRNAs highlighted in blue represent those carrying the 3′ U-tail that is also found in the precursor miRNA. Having found that HCV infection decreases the proportion of miR-122 isomers with a mononucleotide tail (see Fig 3B), we asked whether HCV infection also reprograms the isomer profiles of other liver-resident miRNAs. Further analysis of small RNA sequencing datasets revealed that HCV infection changed the ratios of the mono-A- or mono-U-tailed isomers in a specific set of miRNAs among the top 50 most abundant miRNAs in the liver. The cellular levels of miRNA isomers modified by 3′-end monoadenylation or monouridylation were reduced by >25% upon HCV infection only in a limited number of miRNAs. In only six miRNAs carrying either a single non-templated adenylate residue (miR-122-5p, miR-92a-3p, miR-26a-5p, miR-186-5p, miR-30b-5p, and miR-29a-3p) or a single uridylate residue (miR-122-5p, let-7a-5p, and let-7g-5p have a 3′-end single uridylate residue that is either derived from their precursor forms or added in a non-template-dependent manner; let-7f-5p miR-26a-5p and miR-151a-5p have a non-templated uridylate residue), we observed decreases in their proportions in liver biopsies from patients with HCV. Notably, among these miRNAs, only miR-122 displayed >50% decreases regarding the proportions of all isomers modified by single nucleotide additions.
(TIF)

S5 Fig Analysis of GLD-2 template specificity. Terminal transferase assays were performed with eight different miRNAs (A) randomly selected from the top 50 most abundantly expressed miRNAs in human liver, along with miR-122-5p. Shown below the autoradiogram is radioactivity signal normalized to the template amount (B). In (C), similar analyses were performed with indicated ribonucleotide homopolymers (20-nt).
(TIF)

S6 Fig Effect of the HCV core protein expression on the cellular abundance of GLD-2 and translin. (A) Total cell lysates from the indicated cell lines were analyzed by immunoblotting for GLD-2 and α-tubulin (loading control). (B) Translin mRNA levels in the indicated cell lines were determined by qRT-PCR and normalized to GAPDH abundance. The result represents the mean ± SD from two independent experiments, each involving three technical replicates. “ns” is nonsignificant versus the Huh7/vector cell line control.
(TIF)

S7 Fig Analysis of miR-122 expression in HeLa cells. (A) Total RNA isolated from HeLa and Huh7 cells was analyzed by northern blotting for miR-122. 5S rRNA stained by ethidium bromide was used as a loading control. (B) miR-122 levels in Huh7 and HeLa cells determined by qRT-PCR.
(TIF)

S1 Table Characteristics of liver biopsies used in this study. (TIF)

S2 Table Frequency of RNA types in small RNA sequencing datasets. (TIF)

S3 Table Complete list of all miR-122 sequences obtained by deep sequencing of small RNA libraries from human liver biopsies. (TIF)

S4 Table Profile of miR-122 isomers in Huh7 and Huh7 expressing HCV core protein or infected with HCV. (TIF)
