# Supplementary figures and images for: Hepatitis C Virus Core Protein Promotes miR-122 Destabilization by Inhibiting GLD-2
Source: PLoS Pathog. 2016 Jul 1;12(7):e1005714. doi: 10.1371/journal.ppat.1005714 (PMC4930175; doi:10.1371/journal.ppat.1005714)

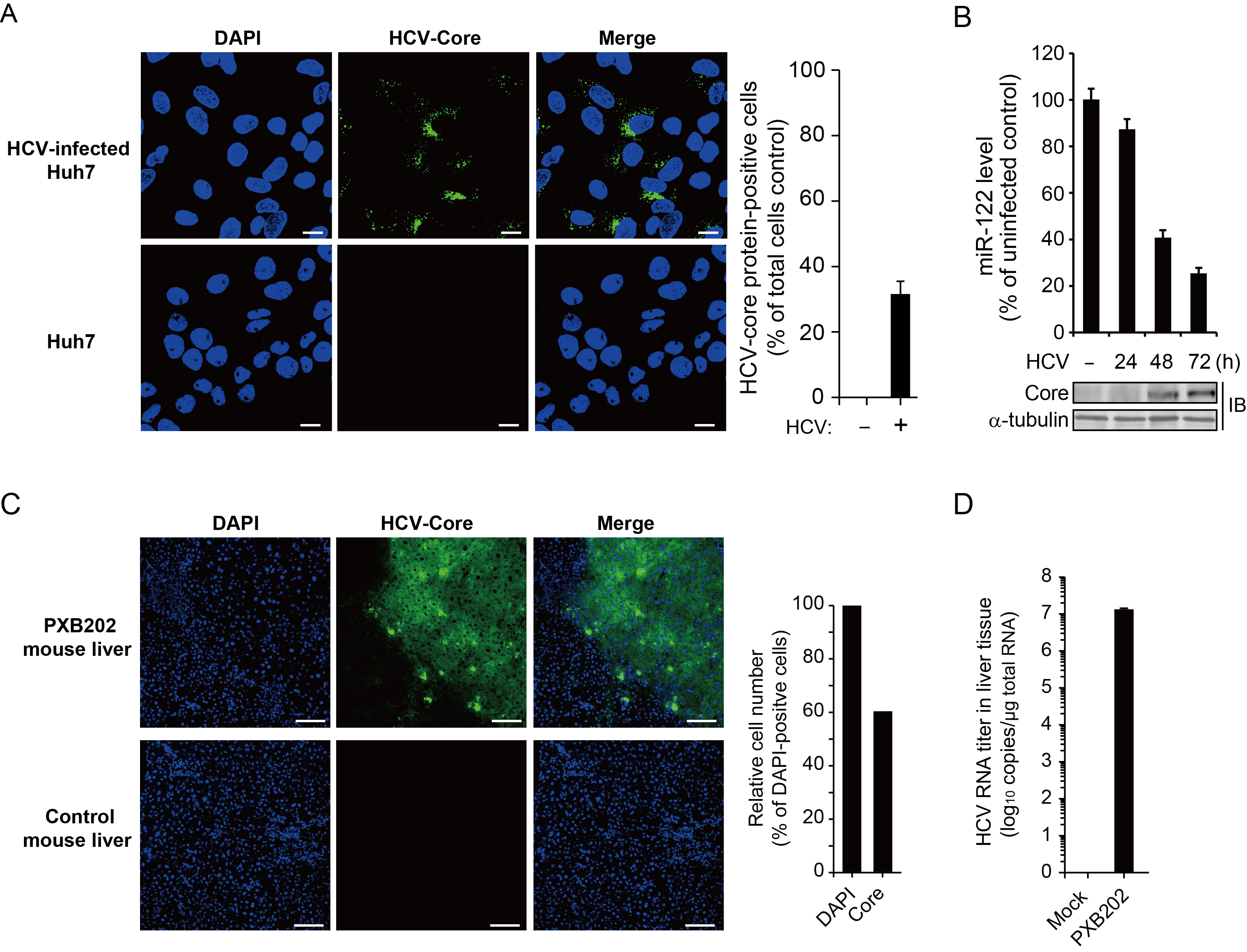

Supplement: S1 Fig — (A) Immunostaining of HCV core protein in HCV-infected Huh7 cells. DAPI, nuclear staining; scale bar, 20 μm. (B) At the indicated time points, miR-122 and HCV core protein levels in HCV-infected Huh7 cells were assessed by RT-PCR and immunoblotting, respectively. (C and D) Analysis of HCV core protein and genome titer in the HCV-infected SCID mice, PXB202 carrying the chimeric liver repopulated with human hepatocytes, by immunostaining (C) and real-time qRT-PCR (D), respectively. The serum HCV titer in PXB202 determined by qRT-PCR was 3 × 10^7^/ml. Scale bar, 100 μm. (TIF) [file ppat.1005714.s001.tif]

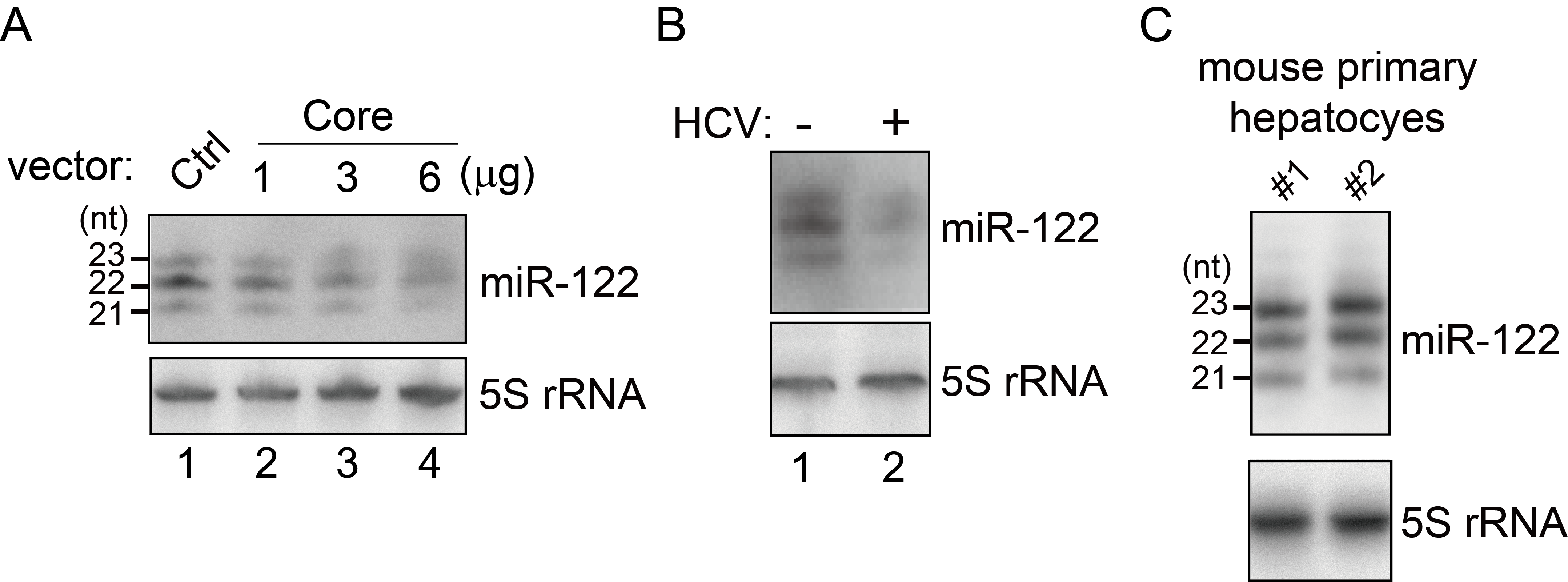

Supplement: S2 Fig — (A) Total RNA from Huh7 cells transfected with pcDNA3.1 (Ctrl) or pcDNA3.1-Flag-core plasmid (Core) were resolved on a 20cm × 20cm denaturing polyacrylamide gel and subjected to northern blotting for miR-122. (B) Huh7 cells infected with HCV at an MOI of 0.25 were analyzed 2 days after infection as described in (A). (C) Detection of miR-122 isomers in primary hepatocytes isolated from mice (#1 and #2). (TIF) [file ppat.1005714.s002.tif]

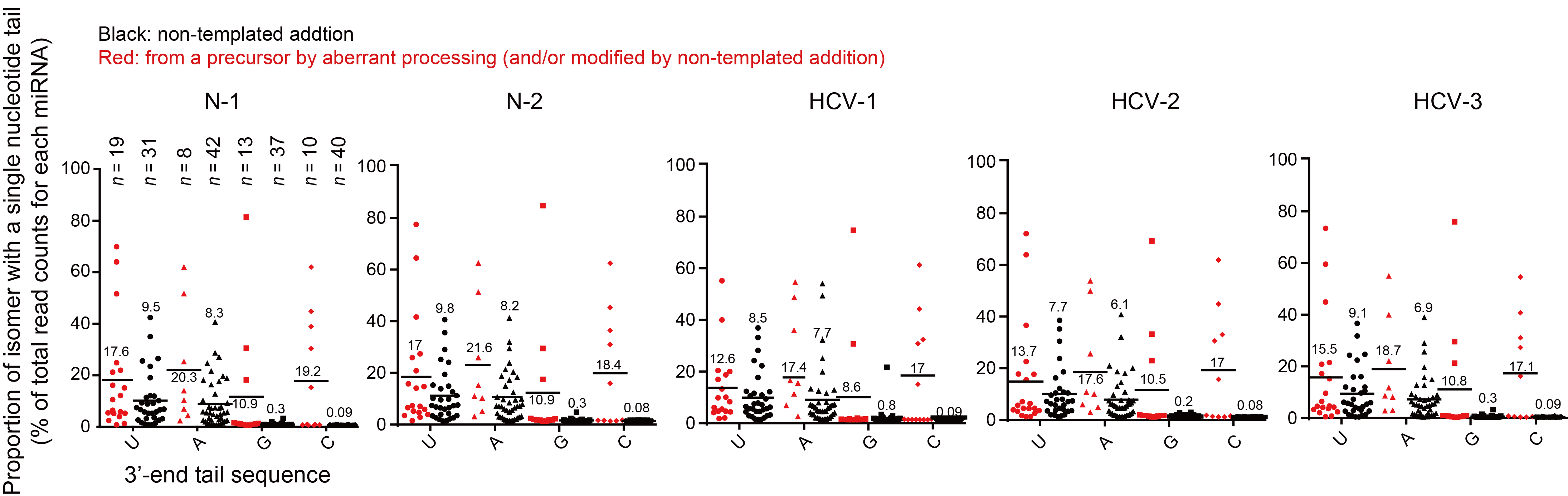

Supplement: S3 Fig — Shown are the percentages of the read count of 3′-terminally single nucleotide-tailed isomers compared with the total read count of each miRNA’s major isomers, which include an isomer with a 3′-end 1-nt deletion, prototype miRNAs, and miRNAs bearing a 3′-terminal single nucleotide tail. N-1 and N-2 denote liver biopsies from healthy controls, and HCV-1 to HCV-3 are liver biopsies from patients with HCV. The numbers above or below the bars indicate the estimated mean value of the proportion for each isomer. miRNA isomers are grouped according to the tail sequence at the 3′ end of individual isomers, which can be either derived from a precursor by aberrant processing and/or by non-templated addition (red symbols) or solely by non-templated addition (black symbols). Via miRNA isomer profile analysis for the top 50 most abundantly expressed miRNAs in liver biopsies, we discovered that these 50 miRNAs, including miR-122, are modified via a non-templated or templated 3′ addition of any of four ribonucleotide residues. The analyzed miRNAs frequently contained a single adenylate or uridylate residue rather than guanylate or cytidylate residues. The average frequencies of mono-U, mono-A, mono-G, and mono-C addition were 12.97%, 9.83%, 2.88%, and 4.07% (when accounting for both templated and non-templated additions), respectively, in the normal liver tissue (N-1). The ratios for mono-G and mono-C additions for these miRNAs were relatively low when considering only non-templated addition. Similar profiles were also observed with another normal liver biopsy N-2 and liver biopsy samples from patients with HCV. These results reveal that miRNA 3′-end modification occurs frequently in liver-resident miRNAs with non-templated monoadenylation and monouridylation being two major modification processes. (TIF) [file ppat.1005714.s003.tif]

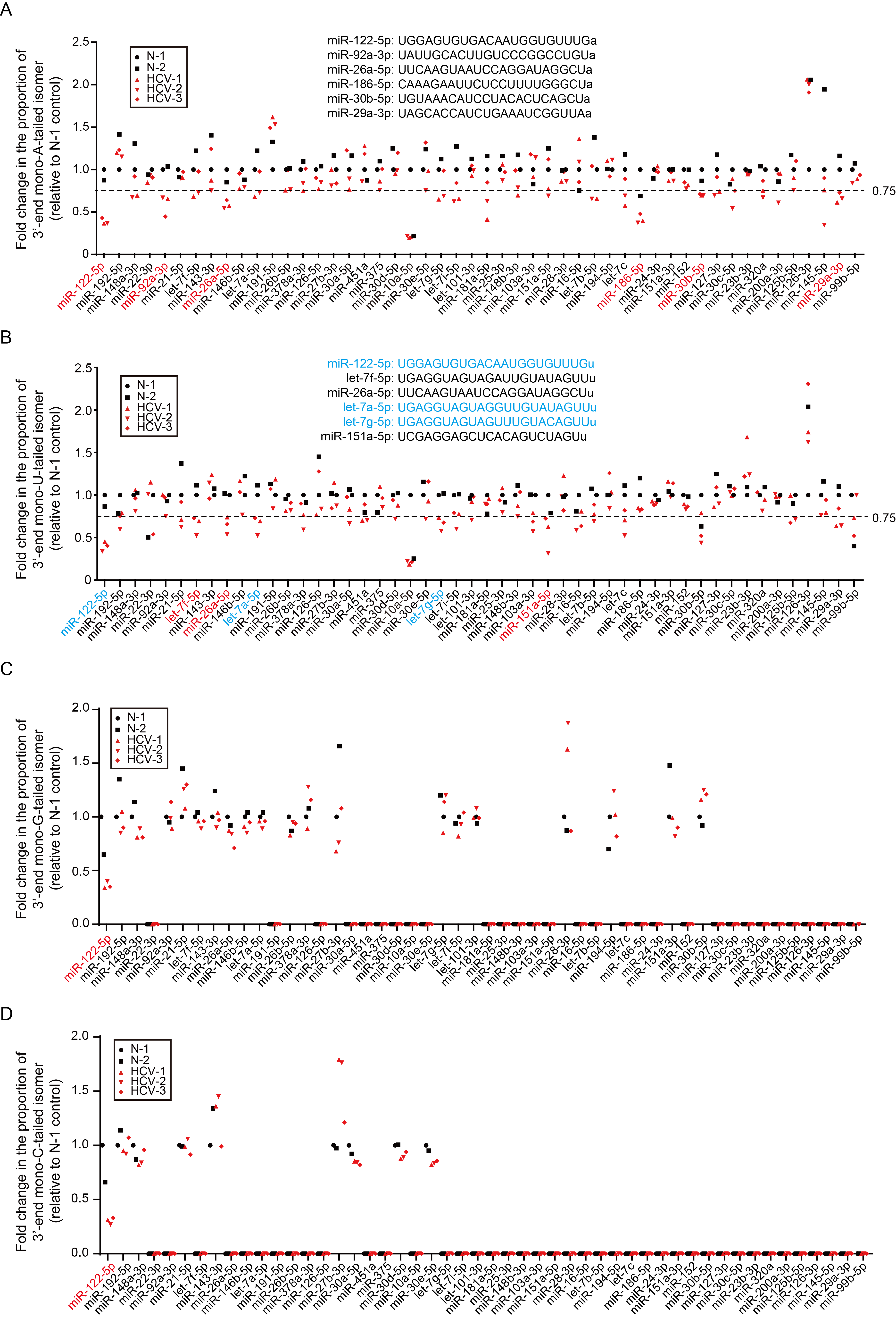

Supplement: S4 Fig — Fold change in the proportion of 3′-end mono-A (A), mono-U (B), mono-G (C), and mono-C (D)-tailed miRNA isomers in liver samples from patients with HCV (HCV-1 to HCV-3, red symbols) compared with the healthy controls (N-1 and N-2, black symbols). Shown are the values for the top 50 most abundant miRNAs present in liver biopsies. miRNAs on the x-axis (starting from miR-122-5p) are in the order of their abundance in liver biopsies. The miRNAs highlighted in red on the x-axis represent those exhibiting >25% decreases in the proportion of the indicated 3′-tailed miRNA isomers in the patient liver biopsies. In (B), the miRNAs highlighted in blue represent those carrying the 3′ U-tail that is also found in the precursor miRNA. Having found that HCV infection decreases the proportion of miR-122 isomers with a mononucleotide tail (see Fig 3B), we asked whether HCV infection also reprograms the isomer profiles of other liver-resident miRNAs. Further analysis of small RNA sequencing datasets revealed that HCV infection changed the ratios of the mono-A- or mono-U-tailed isomers in a specific set of miRNAs among the top 50 most abundant miRNAs in the liver. The cellular levels of miRNA isomers modified by 3′-end monoadenylation or monouridylation were reduced by >25% upon HCV infection only in a limited number of miRNAs. In only six miRNAs carrying either a single non-templated adenylate residue (miR-122-5p, miR-92a-3p, miR-26a-5p, miR-186-5p, miR-30b-5p, and miR-29a-3p) or a single uridylate residue (miR-122-5p, let-7a-5p, and let-7g-5p have a 3′-end single uridylate residue that is either derived from their precursor forms or added in a non-template-dependent manner; let-7f-5p miR-26a-5p and miR-151a-5p have a non-templated uridylate residue), we observed decreases in their proportions in liver biopsies from patients with HCV. Notably, among these miRNAs, only miR-122 displayed >50% decreases regarding the proportions of all isomers modified by single nucleotide additions. (T [file ppat.1005714.s004.tif]

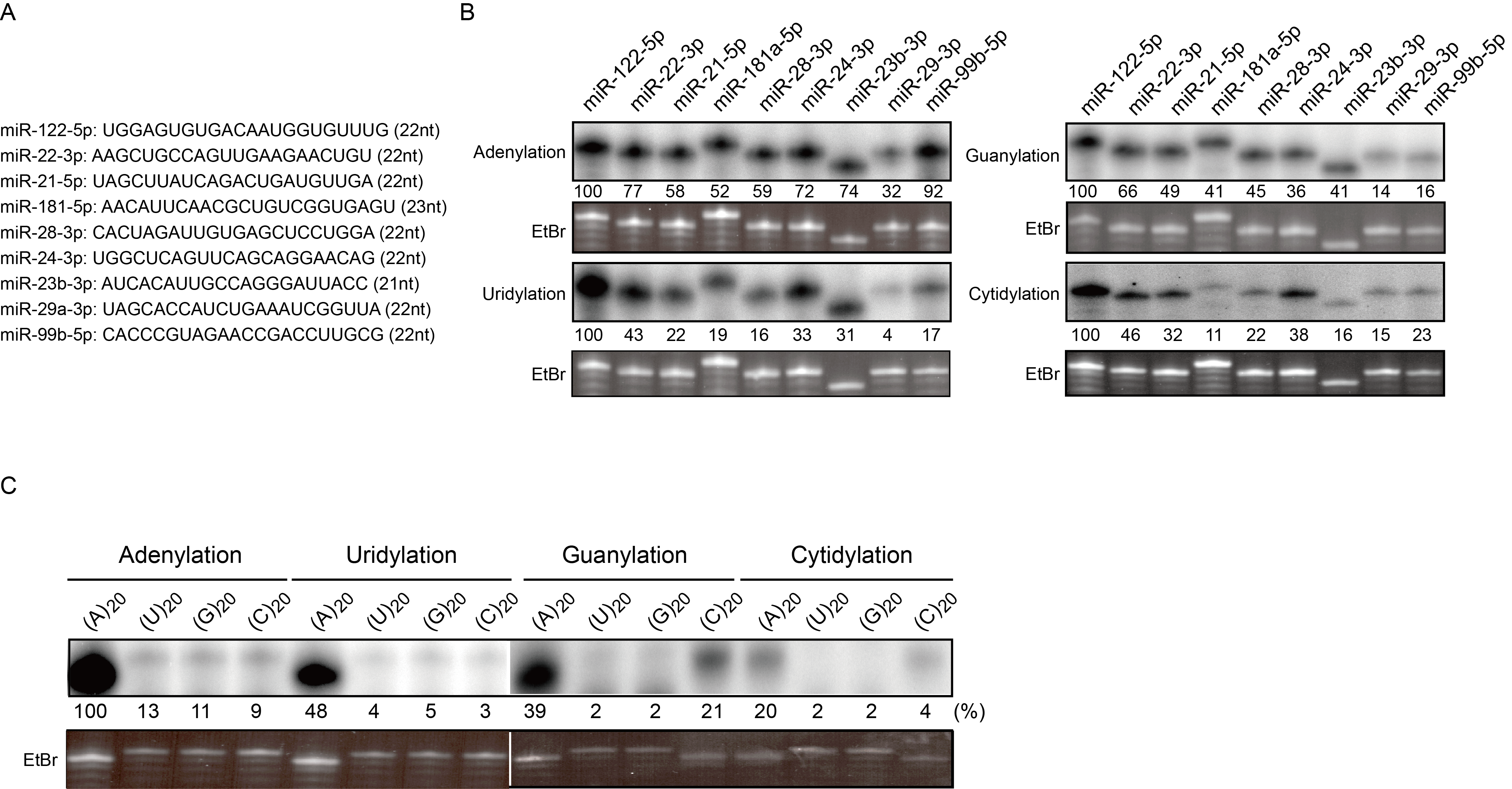

Supplement: S5 Fig — Terminal transferase assays were performed with eight different miRNAs (A) randomly selected from the top 50 most abundantly expressed miRNAs in human liver, along with miR-122-5p. Shown below the autoradiogram is radioactivity signal normalized to the template amount (B). In (C), similar analyses were performed with indicated ribonucleotide homopolymers (20-nt). (TIF) [file ppat.1005714.s005.tif]

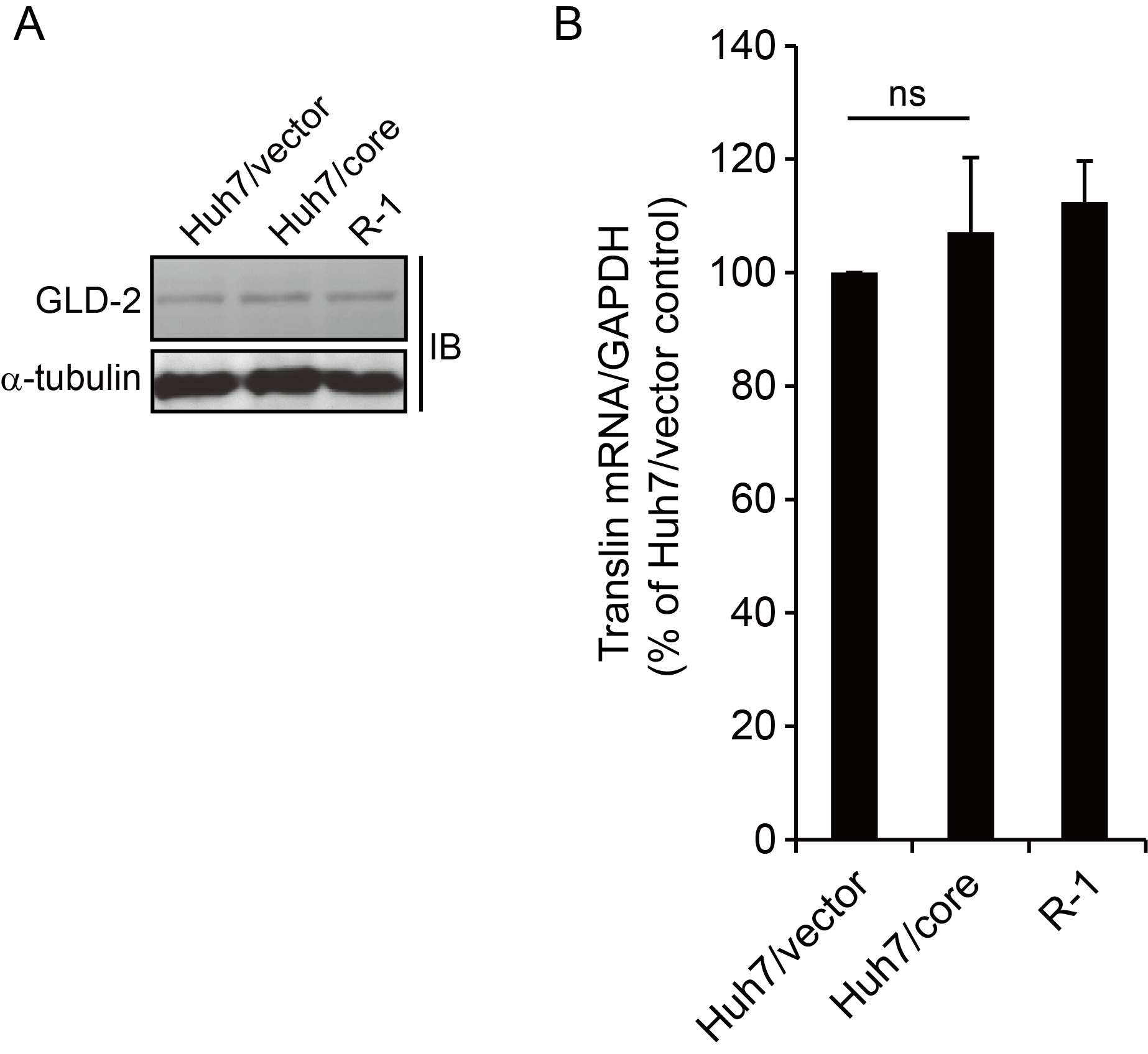

Supplement: S6 Fig — (A) Total cell lysates from the indicated cell lines were analyzed by immunoblotting for GLD-2 and α-tubulin (loading control). (B) Translin mRNA levels in the indicated cell lines were determined by qRT-PCR and normalized to GAPDH abundance. The result represents the mean ± SD from two independent experiments, each involving three technical replicates. “ns” is nonsignificant versus the Huh7/vector cell line control. (TIF) [file ppat.1005714.s006.tif]

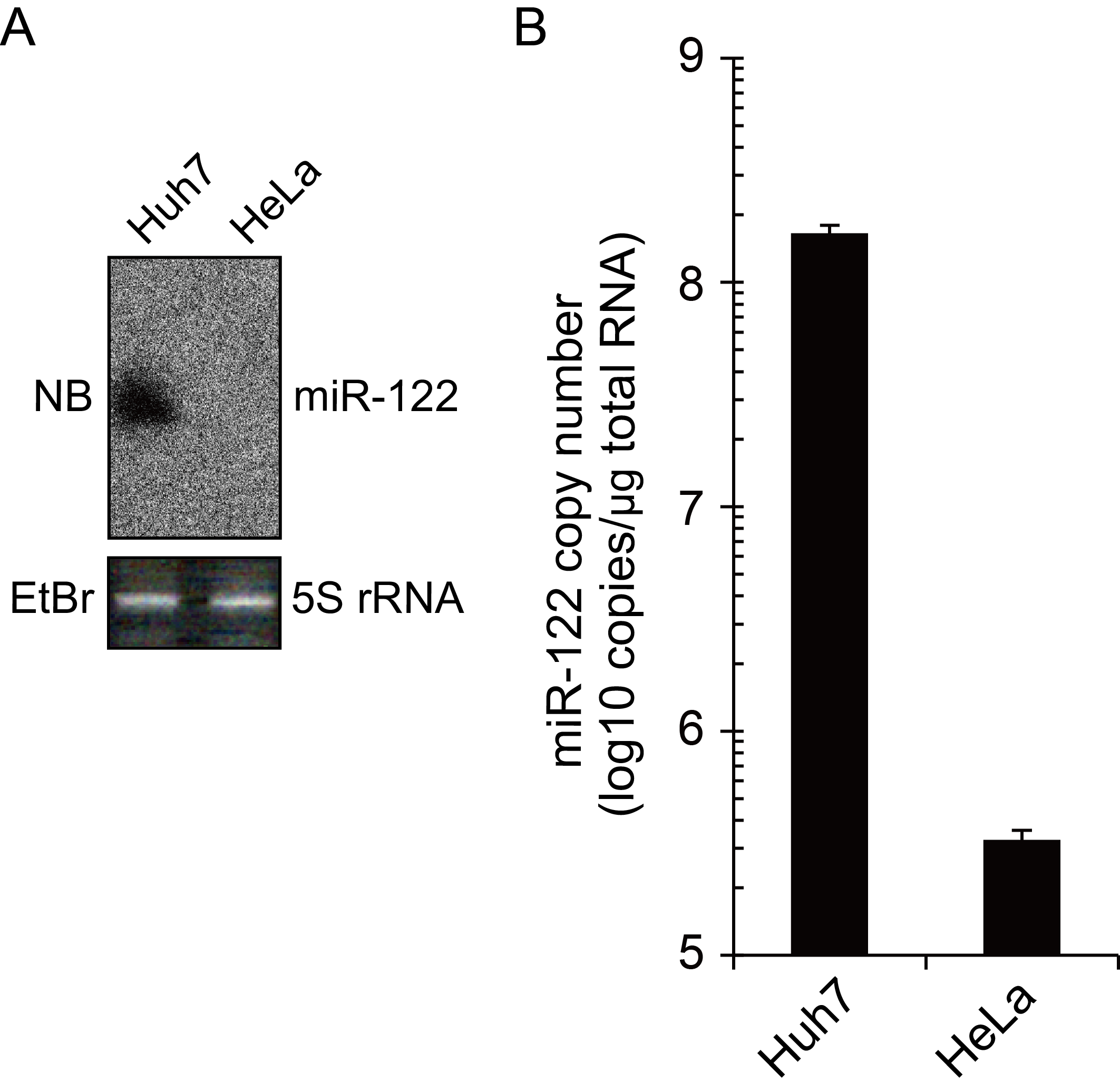

Supplement: S7 Fig — (A) Total RNA isolated from HeLa and Huh7 cells was analyzed by northern blotting for miR-122. 5S rRNA stained by ethidium bromide was used as a loading control. (B) miR-122 levels in Huh7 and HeLa cells determined by qRT-PCR. (TIF) [file ppat.1005714.s007.tif]

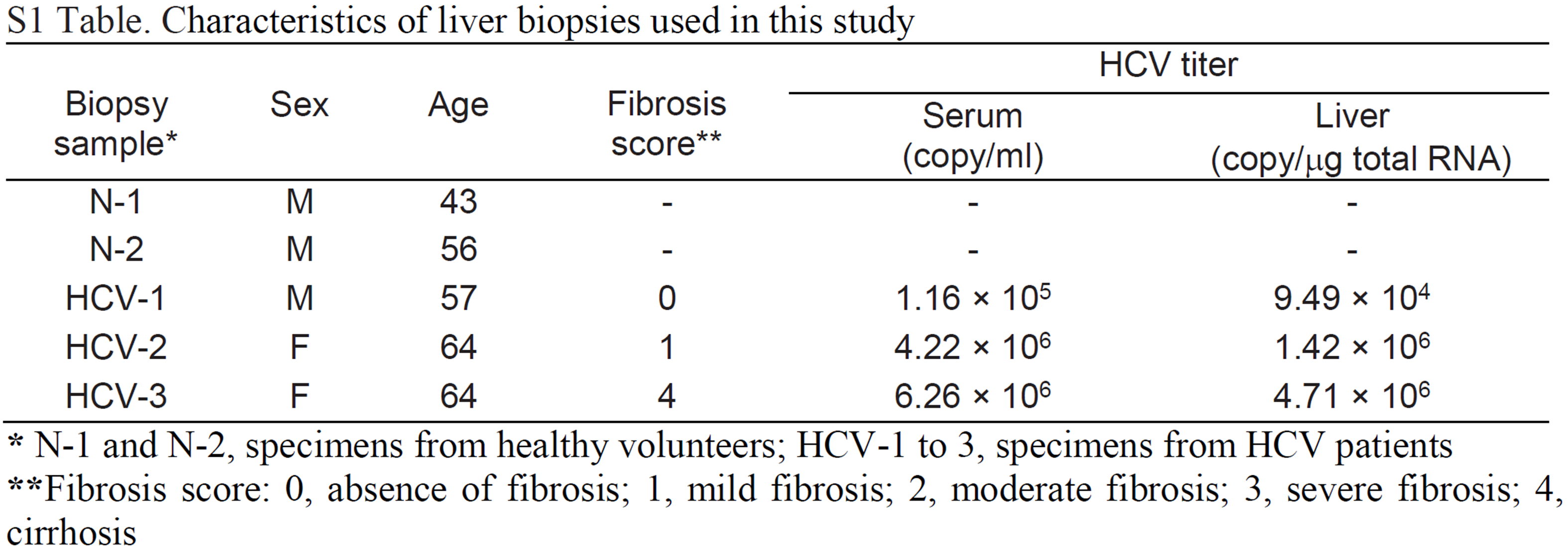

Supplement: S1 Table — (TIF) [file ppat.1005714.s008.tif]

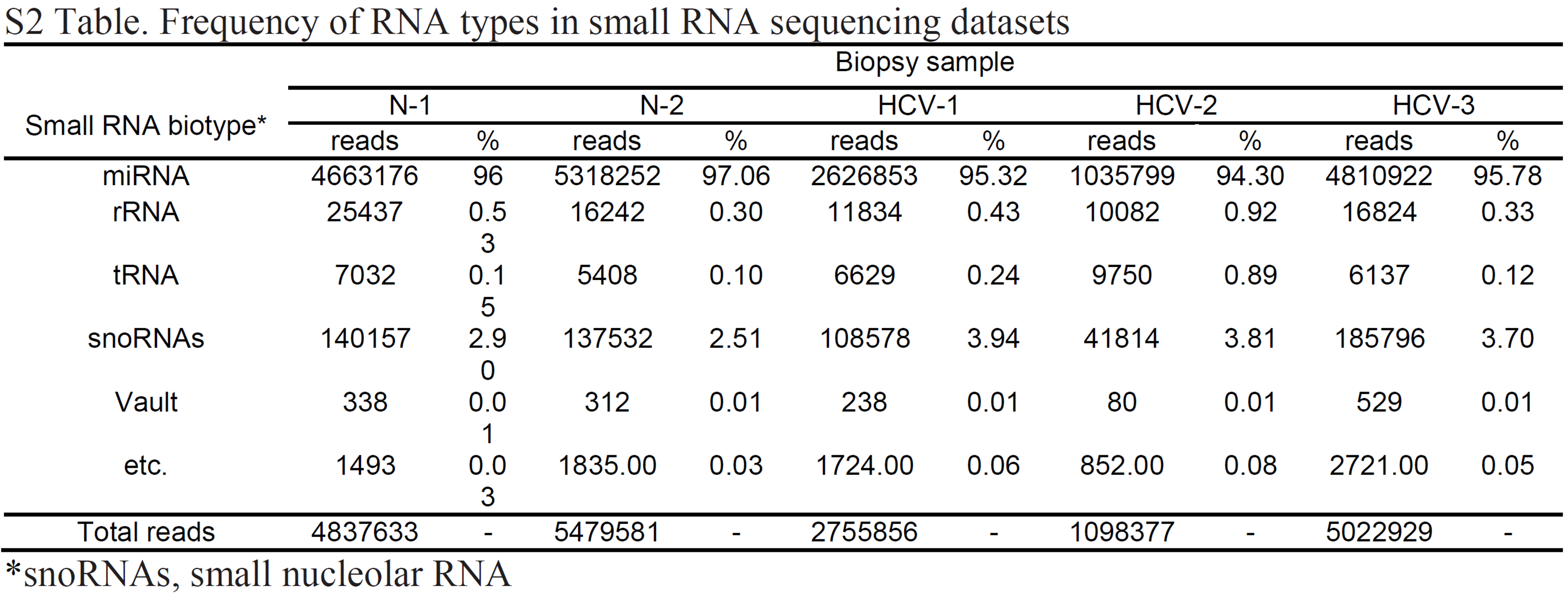

Supplement: S2 Table — (TIF) [file ppat.1005714.s009.tif]

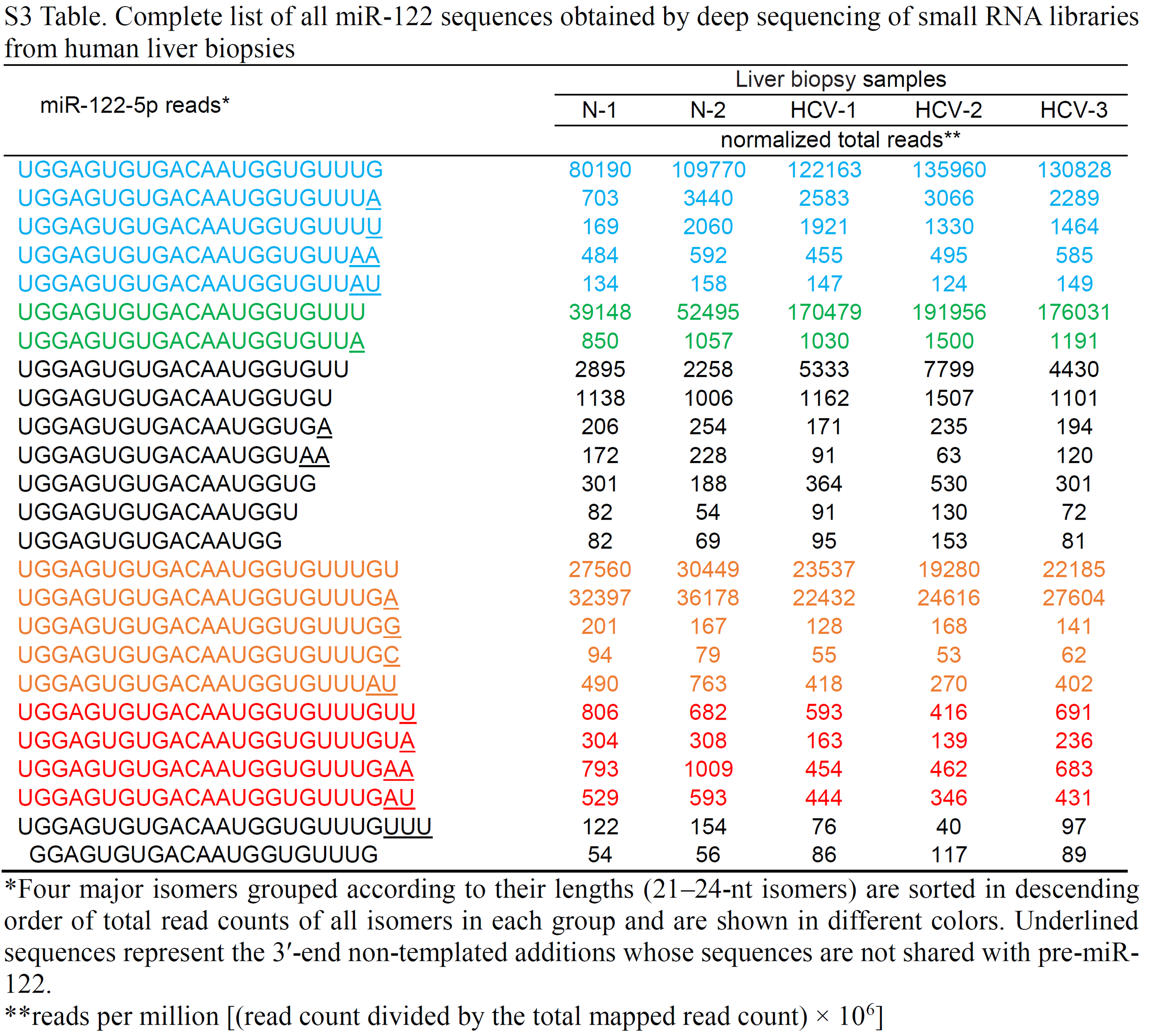

Supplement: S3 Table — (TIF) [file ppat.1005714.s010.tif]

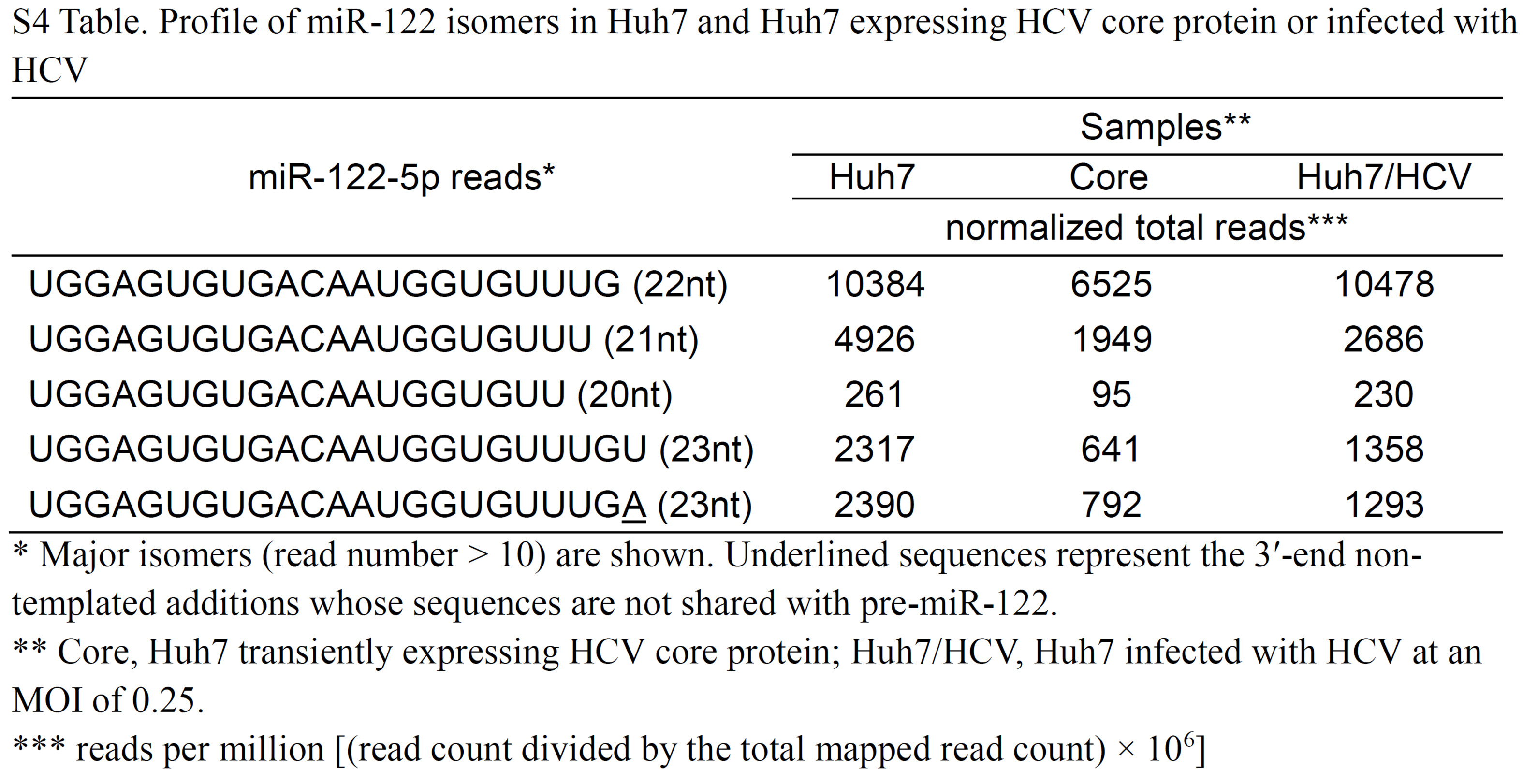

Supplement: S4 Table — (TIF) [file ppat.1005714.s011.tif]
